# Models of Self-Peptide Sampling by Developing T Cells Identify Candidate Mechanisms of Thymic Selection

**DOI:** 10.1371/journal.pcbi.1003102

**Published:** 2013-07-25

**Authors:** Iren Bains, Hisse M. van Santen, Benedict Seddon, Andrew J. Yates

**Affiliations:** 1 Immune Cell Biology, MRC National Institute for Medical Research, Mill Hill, London, United Kingdom; 2 Department of Systems and Computational Biology, Albert Einstein College of Medicine, New York, New York, United States of America; 3 Centro Biologia Molecular Severo Ochoa, CSIC/Universidad Autonoma de Madrid, Madrid, Spain; 4 Department of Microbiology and Immunology, Albert Einstein College of Medicine, New York, New York, United States of America; Massachusetts Institute of Technology, United States of America

## Abstract

Conventional and regulatory T cells develop in the thymus where they are exposed to samples of self-peptide MHC (pMHC) ligands. This probabilistic process selects for cells within a range of responsiveness that allows the detection of foreign antigen without excessive responses to self. Regulatory T cells are thought to lie at the higher end of the spectrum of acceptable self-reactivity and play a crucial role in the control of autoimmunity and tolerance to innocuous antigens. While many studies have elucidated key elements influencing lineage commitment, we still lack a full understanding of how thymocytes integrate signals obtained by sampling self-peptides to make fate decisions. To address this problem, we apply stochastic models of signal integration by T cells to data from a study quantifying the development of the two lineages using controllable levels of agonist peptide in the thymus. We find two models are able to explain the observations; one in which T cells continually re-assess fate decisions on the basis of multiple summed proximal signals from TCR-pMHC interactions; and another in which TCR sensitivity is modulated over time, such that contact with the same pMHC ligand may lead to divergent outcomes at different stages of development. Neither model requires that T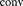 and T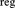 are differentially susceptible to deletion or that the two lineages need qualitatively different signals for development, as have been proposed. We find additional support for the variable-sensitivity model, which is able to explain apparently paradoxical observations regarding the effect of partial and strong agonists on T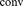 and T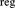 development.

## Author Summary

T cells develop in the thymus, where they are vetted – they must respond weakly to self-antigens, but not so strongly as to risk causing autoimmunity. This selection process involves developing T cells being exposed to a large sample of self-peptides presented on specialised cells in the thymus, and deciding to die or to differentiate into mature T cells of either conventional or regulatory lineages. The rules by which T cells assimilate information from these interactions to make these decisions are not known. In this study we use previously published data to assess and discriminate between different models of thymic selection and find the most support for a model in which T cells vary their sensitivity to self-peptides during their development. This allows fate decisions to be made on the basis of as few as one peptide at a time, which allows for fine specificity in the selection process.

## Introduction

Conventional 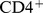 T cells (T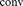) and 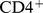 T regulatory cells (T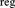) are essential components of the adaptive immune system. Conventional T cells develop effector function in response to foreign antigens, while natural T regulatory cells produced in the thymus play a key role in the maintenance of tolerance to self-antigens and prevent autoimmune diseases (reviewed in, for example, [1]). Both populations are derived from precursors in the thymus that develop, undergo selection and differentiate into different T cell lineages. The differentiation of a thymocyte into the mature 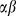 T cell repertoire is dependent on the engagement of its T cell receptor (TCR) with endogenous peptides presented by major histocompatibility complex (MHC) molecules on thymic antigen presenting cells. Continued very weak or null interactions between the TCR and peptide-MHC ligands (pMHC) lead to failure to positively select (‘death by neglect’) while excessively strong TCR-pMHC interactions lead to negative selection, removing highly autoreactive cells from the T cell repertoire. However, the precise rules underlying T cell precursor fate are not well understood; based on its exposure to a sample of pMHC, how and when does a thymocyte decide to become a T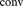, a T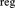, or be deleted?

Studies using fetal thymic organ cultures have shown that there exists a sharp avidity threshold between positive and negatively selecting ligands [2], [3]. There is substantial evidence indicating that T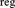 are induced by TCR signals that lie below this negative selection threshold, but above that required for selection into the conventional T cell pool [4]–[8]. However, many uncertainties remain. It has been shown that expression of cognate antigen (which we loosely refer to ‘agonist peptide’) in the thymic epithelium is required for the generation of T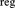 [9]–[13], but a recent study showed that T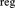 commitment occurs over a wide range of TCR affinities for a ubiquitously expressed self antigen [14]. Further, the partitioning of fates with increasing strength of recognition for self (deletion
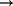
T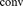 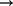
T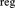 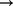
deletion) appears to be questioned by a study in which both expression of an agonist and a weaker partial-agonist could enhance deletion, but only the agonist was able to induce the formation of 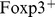 regulatory T cells [15], suggesting that either the mapping of avidity to fate is more complex or that qualitatively different signals are required for T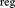 and T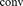 selection.

Many experimental models using TCR transgenic cells (clonal populations of T cells with identical TCR) have shown that these cells can develop into both the regulatory and conventional lineages together in the same environment. This observation implies that there is stochasticity in fate determination. This stochasticity can be partitioned conceptually into two sources that are not mutually exclusive. First, there may be heterogeneity at the early double positive 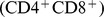 stage of development, even within a clonal population, that pre-disposes cells to different fates. This heterogeneity might derive, for example, from differences in expression of factors determining the baseline levels or dynamic range of TCR signalling, or other signalling proteins related to lineage commitment. Second, stochasticity may be present later in the selection process, arising at least in part because each thymocyte encounters an independent sample of self-peptide ligands. Evidence for the latter comes from observations that probabilities of deletion and T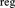 generation have been shown to vary with levels of agonist-peptide expression; *in-vivo* studies in TCR transgenic mice [16]–[18] and *in-vitro* fetal thymic organ culture [19]–[21] have shown that the efficiency of T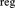 selection increases with modest increases in agonist-peptide expression, but drops when expression is high. (We use the term *efficiency* here interchangeably with the probability of experiencing a given fate.) The efficiency of selection into the T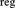 lineage also decreases in the presence of increasing numbers of cells of the same specificity [14], [15], [22]–[24]. Thus the availability of relevant ligands, either in absolute terms or through competition, can influence fate decisions.

The timing of an interaction with a ligand may also influence fate. There is evidence that the sensitivity of thymocytes to TCR stimulation is increased during maturation through the subcellular localisation of signalling molecules such as tyrosine kinase Lck [25]; the inhibition of extracellular signal-regulated kinase (ERK) activation and increased expression of inhibitory tyrosine phosphatase SHP-1 [26]; the upregulation of the negative regulator CD5 [27]; and the increased expression of ZAP-70, a downstream target of TCR signalling [28]. However, the expression of miR-181a, a microRNA that enhances sensitivity to TCR stimulation, is reduced during thymic development [29], [30], and TCR signalling in response to low-affinity pMHC ligands is strongest in immature thymocytes [31]. The net effect of changes in TCR signal activating and inhibiting factors is not clear, but it is possible that stimulation with the same ligand will lead to different levels of activation in the same thymocyte at different stages of development.

The challenge of synthesising these observations and describing quantitatively how the affinity, number and timing of pMHC contacts shape the developing T cell repertoire invites a mathematical modelling approach. Models of thymic selection have been successful in providing insight into the relationship between diversity of self peptides sampled in the thymus and the cross-reactivity [32], [33], alloreactivity [34], size [35], [36] and CD4SP/CD8SP ratio [37] of the selected repertoire. Models have also helped us understand the relation of HLA phenotype to viral epitope recognition [38] and the trade-off between MHC and T cell receptor diversity [39]. In this study we use stochastic (probabilistic) models to describe previously published *in vivo* data describing T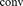 and T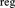 commitment of a transgenic cell population in the presence of varying densities of agonist peptide in the thymus [16]. These data allow us to test and discriminate between models of how developing thymocytes might integrate signals received from pMHC ligands to make lineage decisions.

Models of thymic selection must relate the physical interaction between a TCR and a pMHC ligand to the signal interpreted or integrated by the thymocyte. There is evidence to support competing models of TCR-pMHC interactions in which the level of T cell activation is determined by TCR-pMHC dwell times, through the kinetic proofreading model [40], [41], TCR occupancy [42]–[44] and overall pMHC ligand affinity [45]. Previous approaches to quantitative modelling of TCR-pMHC interactions can be divided into three broad categories: (i) detailed modelling of signal transduction immediately downstream of TCR-pMHC engagement [46], [47]; (ii) kinetic models of binding events using measured rates of TCR-pMHC association and disassociation [48], [49]; and (iii) the use of a ‘string model’ framework in which the strength of an interaction is determined by pairwise interaction energies between peptides and the aligned residues of amino acids on the variable CDR3 loop of randomly generated TCRs [32], [33]. However, binding kinetic parameters are not available for the full range of endogenous peptides that are encountered during thymic development, and uncertainty remains in the relation between avidity and the signalling thresholds determining fate decisions. Here, we abstract from the mechanistic model of signal strength derived from molecular interactions. Instead we assume a distribution of signal strengths that a given TCR derives from pMHC ligands, in which low-strength signalling events occur with the greatest likelihood and, in line with our knowledge of the specificity of T cell recognition, stronger signalling events occur with decreasing probability. We show that our conclusions are insensitive to the precise form of this distribution.

We explore candidate mechanisms of T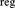 and T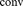 selection using the canonical hypothesis that signals associated with T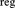 commitment are stronger than those required for T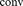 commitment but are below the threshold for negative selection. We use the data of van Santen *et al.* [16] to reject a simple model of the selection process in which thymocyte fate is based on testing sequential single TCR-pMHC interactions. Instead, we find the data can be explained with two generalisations of this model in which perceived TCR signal strength correlates to a strict hierarchy of cell fates (neglect
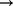
T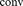 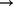
T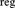 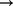 negative selection). In both models, thymocytes are continuously initiating fate decisions based on measuring the strength of binding to self peptide-MHC ligands in a series of encounters with antigen-presenting cells. In one class of model, the 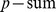 model, cells measure the avidity of each encounter, each of which comprises binding to a sample of multiple self-peptide-MHC ligands simultaneously. The 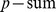 model is motivated by studies implicating the integration of signals from multiple pMHC interactions in the priming of mature T cells by antigen [50]–[53]. In the second class of model, the two-phase model, we examine the consequences of TCR sensitivity of thymocytes varying during development. In this model a cell's interpretation of the signal derived from a given ligand depends on whether it occurs early or late in selection. We show that both models are able to describe the data, and also make predictions that are consistent with studies quantifying the efficiency of T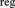 selection with avidity [14]. However, we argue that variable TCR sensitivity is required to explain the effect of partial and full agonist peptide expression in the thymus on T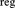 generation and negative selection reported by Cozzo Picca and colleagues [15].

## Methods

### Experimental data

**Figure 1:**
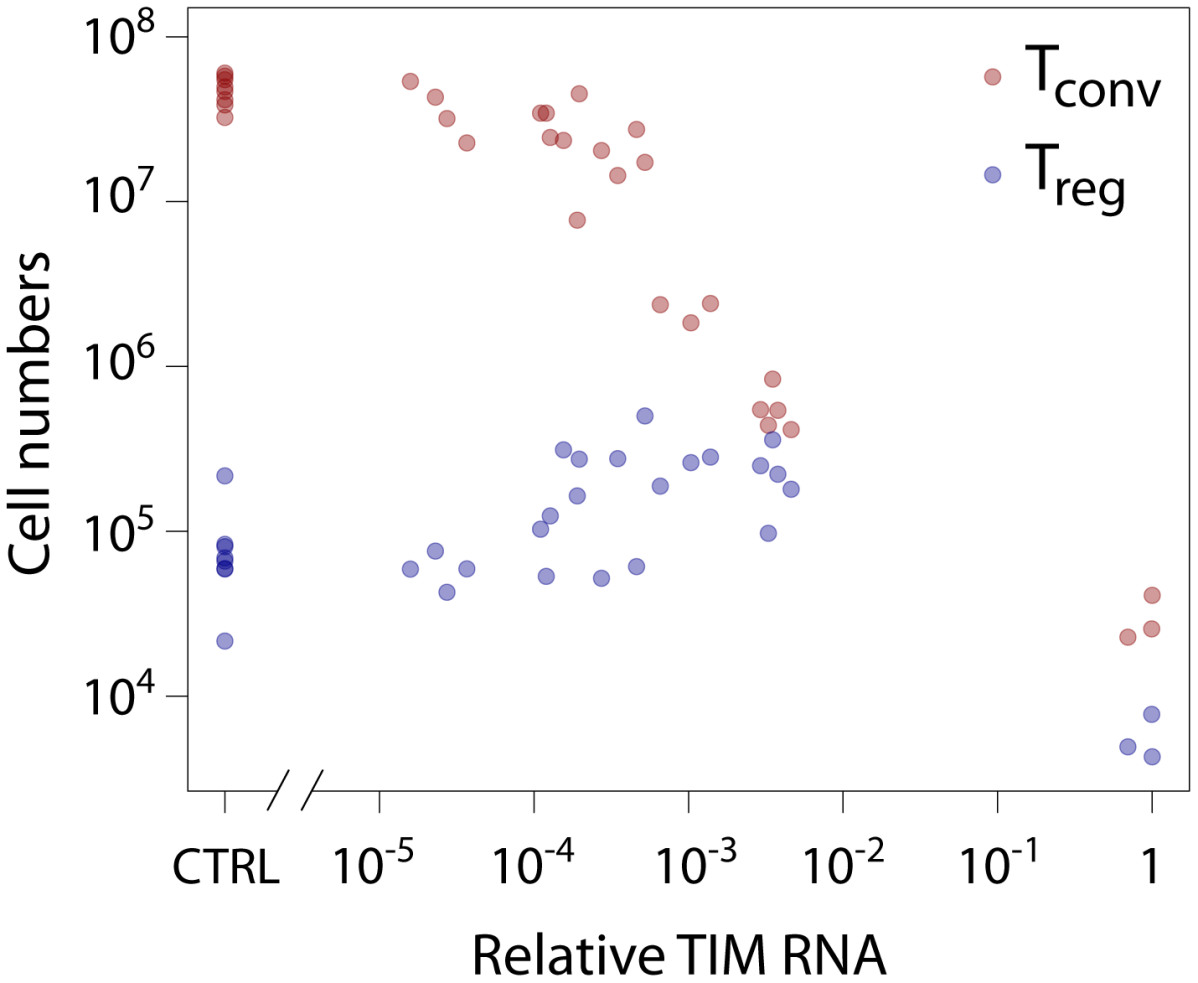
Data taken from van Santen *et al.* [16]. Absolute number of clonotype positive 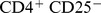 (T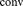, red circles) and 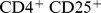 (T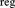, blue circles) thymocytes in tetracycline-treated TAND mice, as a function of the relative expression level of TIM RNA in the thymus of these animals. Control animals lacked either the transactivator or reporter transgene.

We use data from van Santen *et al.* (2004) [16] in which the frequency of high affinity intra-thymic ligands was manipulated *in vivo*. Briefly, a mouse line was used that employs the tetracycline inducible system to conditionally express an invariant chain mutant, bearing the T cell epitope from moth cytochrome *c* (MCC) in place of the class-II associated invariant chain peptide (CLIP)-encoding region (TIM). TIM was expressed in both cortical and medullary thymic epithelial cells (cTEC and mTEC), and at controllable and graded levels. The mice also contained a transgene encoding a TCR specific for this peptide, such that in the absence of induced TIM, these cells differentiated efficiently into mature 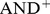 CD4 single positive thymocytes. Expression of TIM, measured by TIM RNA transcripts via real-time PCR, influenced both T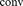 and T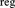 formation in a non-linear fashion (Figure 1).

In Figure 1 we see that (i) low frequencies of a strong agonist (TIM) do not affect the selection of TCR-specific (AND) thymocytes into the conventional T cell pool; (ii) moderate increases in agonist expression lead to increasing efficiency of selection of AND cells into T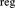 (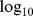
(T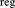) against 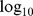
(Relative TIM RNA) between 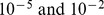; Pearson correlation 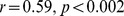) and a concurrent drop in the efficiency of T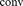 selection; and (iii) high frequencies of a strong agonist lead to the deletion of AND T cells. A very similar trend was observed by Cozzo Picca *et al.* [17] using TCR transgenic cells specific for an epitope of influenza virus in the presence of different levels of expression of this agonist. Atibalentja *et al.* [18] also observed this trend following intravenous injection of varying concentrations of hen egg-white lysozyme (HEL), which was rapidly processed and presented in the thymus, resulting in the negative selection of specific TCR transgenic T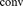 and an increase in TCR transgenic T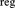 at low, but loss at higher, HEL concentrations.

### Mathematical models

**Figure 2:**
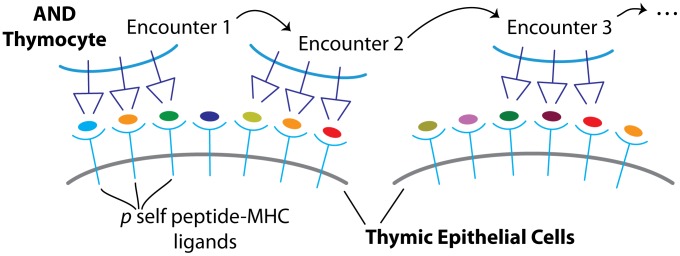
Thymocyte encounters with self-peptides. An *encounter* is defined as the simultaneous or temporally proximal binding of 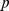 TCR to 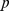 pMHC ligands on a thymic epithelial cell. Here, 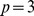.

Developing thymocytes survey pMHC ligands presented on the surface of thymic epithelial cells. In all models we assume that fate decisions are continually reassessed based on ‘encounters’, each of which is the sum of 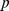 interactions with pMHC (Figure 2), where 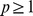. Each thymocyte participates in 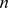 encounters at most, where a thymocyte might undergo negative selection, or initiate development into the T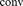 or T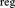 lineages, before reaching its 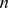-th encounter. We assume that each encounter with one or more pMHC can be divided into four categories determined by its affinity or avidity and the resulting signal through the T cell receptor(s). These are (i) a weak or null signal below that required for positive selection; (ii) a signal sufficient for selection into the T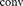 lineage; (iii) a signal that initiates selection into the T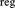 lineage; and (iv) a strong signal that leads to deletion.

We considered two classes of models. In one, the distribution of signal strengths resulting from encounters is constant throughout the selection period - the 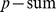 model. In the other, the two-phase model, we allow for the possibility that this distribution shifts during selection as a result of temporal changes in TCR sensitivity.

#### Fate decisions made by integrating TCR signals; the 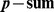 model

The 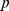 interactions constituting an encounter may occur simultaneously, or sequentially within a time interval that is short compared to the decay time for TCR signals transduced by binding to pMHC. We consider an encounter to be the unit of information that can influence fate decisions. When one TCR binds to one randomly chosen pMHC, the contact results in a signal of strength drawn from an unknown probability distribution. One correlate of ‘strength’ might be the affinity of binding. Indeed affinity of binding to selecting pMHC ligands has been demonstrated to be linearly proportional to selection efficiency [14]. Similarly, when signals from multiple, proximal TCR-pMHC binding events are integrated in each encounter (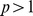), the resulting signal strength might be related to the avidity of the interaction. However we allow freedom in the interpretation of the term *strength* to allow for non-linear relationships between the off-rate of a TCR-pMHC complex and the signal transduced by the TCR. It is simply the quantity resulting from each encounter that the T cell uses in its fate-determination machinery. We assume a log-normal distribution of signal strengths, for reasons we discuss below.

**Figure 3:**
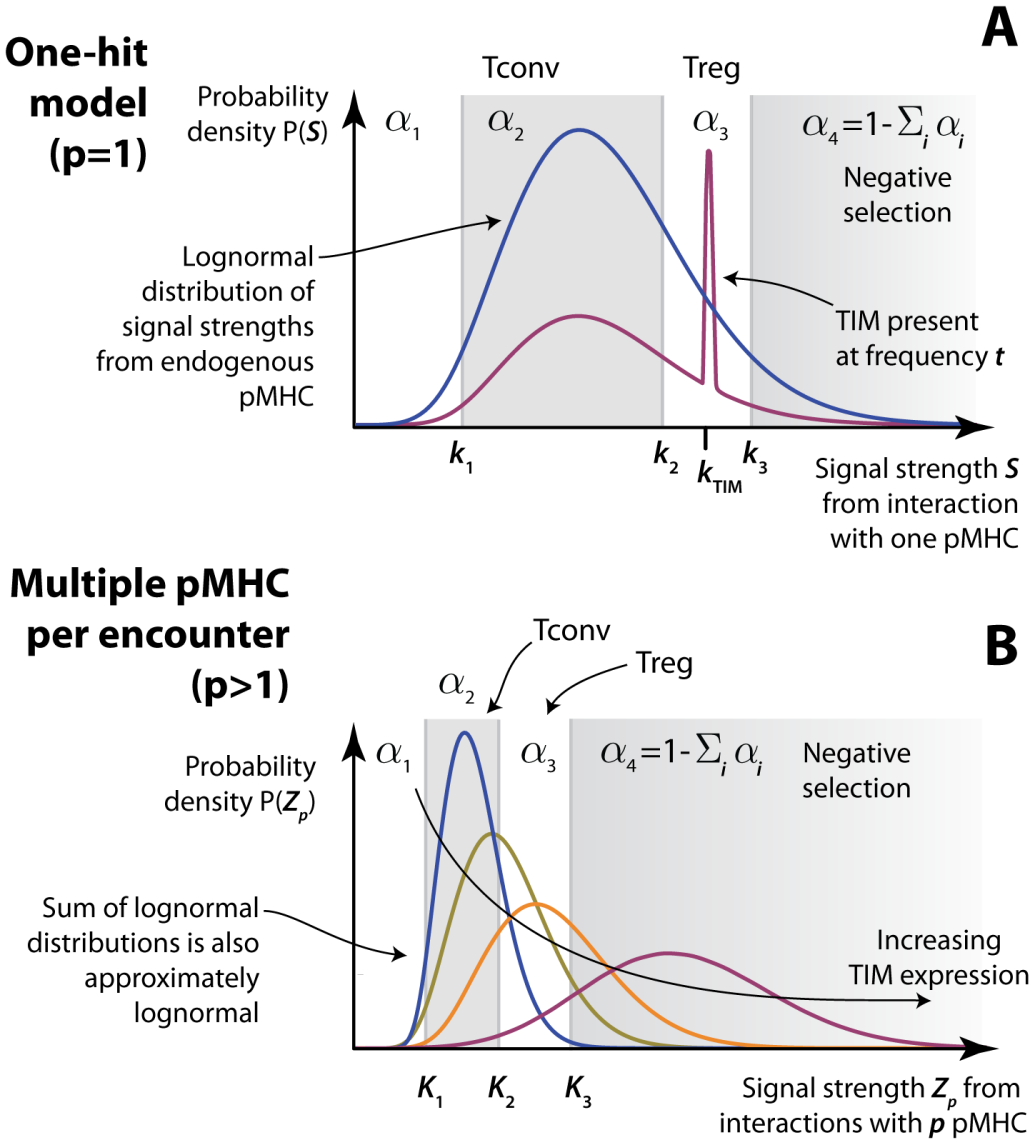
The influence of TIM expression on the distribution of signal strengths resulting from thymocyte encounters. **A**: The case 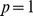. The probabilities 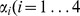) are those of a signal lying within the different selecting regions. Blue curve; log-normally distributed signal strengths 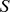 from self pMHC in the absence of TIM expression. Area under the curve = 1. Red curve; TIM expressed at frequency 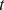 superimposed on this wild-type (endogenous pMHC) distribution. The spike at 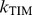 (shown for convenience here with finite width and height) is a point mass in the probability distribution, of area 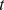; the remainder is of area 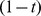. **B**: For 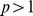 the different selecting regions lie beween different signal strength thresholds 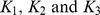. Increasing TIM expression 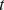 (the proportion of pMHC within an encounter, on average) shifts the distribution of signal strengths rightwards.

To illustrate the calculation of selection efficiencies in this model, consider the case 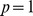 (Figure 3A). Selection into the conventional T cell lineage requires:

1. at least one encounter with strength greater than a positive selection threshold, 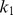;
2. all 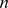 encounters below a higher threshold 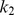.

Experimental evidence suggests that T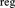 development requires agonist peptide to be presented in the thymus [9], [10], [12], [24], [54]. The canonical explanation is that T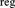 are induced by TCR signals that lie below the threshold of negative selection, but above that required for selection into the conventional T cell pool. So we define a negative selection threshold 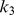, above 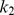, such that an encounter between 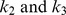 triggers divergence into the T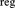 lineage. For T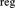 selection, then,

1. at least one of the 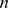 encounters is above a positive selection threshold, 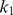 (to pass positive selection),
2. all 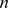 encounters are below the threshold 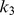 (to avoid negative selection),
3. at least one encounter occurs between thresholds 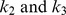.

While this model does not contain time explicitly, the 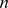 encounters are considered to occur sequentially and so negative selection (deletion) can be initiated at any time by an encounter with strength 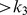. This also means that it is possible, for example, for a cell to receive a signal within the T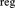 region and initiate development into that lineage, but later to have an encounter above 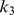 and be deleted. It also means than 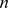 is an upper limit on the number of thymocyte encounters; the mean number of encounters will be fewer than 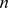 due to early termination through negative selection.

We then calculate the probability of each fate (fail positive selection, T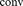, T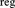, deletion) after 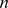 encounters. These depend simply on the probabilities 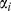 of an encounter falling in each region (Figure 3A, blue curve);
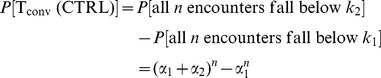
(1)

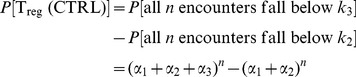
(2)


Now assume a proportion 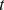 of endogenous peptides are replaced by the agonist peptide TIM. Each TCR-pMHC contact will involve an endogenous peptide with probability 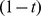 or TIM with probability 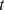. The signal strength derived from this contact will respectively be lognormally-distributed or with fixed strength 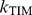. Agonist ligands appear to induce deletion as well as T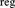 commitment [9]–[13], [16] and so it is likely that 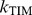 lies above 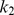. To illustrate, assume it lies within the window that triggers T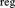 commitment, the region bounded by 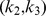. At expression level 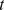, the probabilities 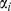 change as follows (Figure 3A, red curve):
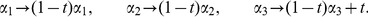
(3)For any 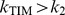, the probability of selection into T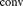 is
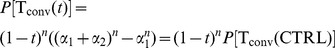
(4)and the probability of selection into T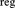 is
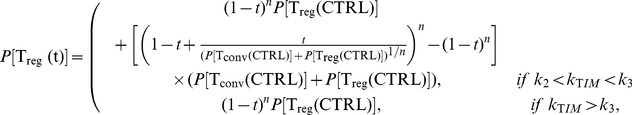
(5)which is a function of the maximum number of encounters 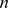 but is independent of the selection thresholds or the probabilities 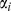.

When 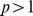, we assume each encounter is of strength 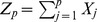. The 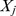 are identically distributed random variables representing the strength of a single TCR-pMHC binding. Each binding generates either (i) a signal arising from a randomly selected endogenous pMHC, with probability 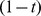, or (ii) a signal of strength 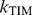 resulting from an interaction with TIM pMHC with probability 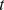. We denote the selection thresholds as 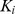. When 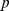 is small, the distribution of signal strengths contains point masses at 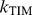, 2 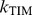, and so on. As 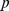 increases, the distribution becomes smoother and shifts rightwards with increasing 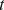 (Figure 3B). Text S1 contains the calculation of the selection probabilities for 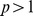.

Given the complexities of TCR signalling, individual TCR binding events may not contribute linearly to an encounter's strength, however ‘strength’ is defined. However for our arguments all we require is (i) that endogeneous pMHC provide a smooth distribution of signal strengths arising from encounters, (ii) when agonist is present at frequency 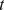, this distribution shifts rightwards, and (iii) the more pMHC involved in an encounter, the smoother this perturbed distribution is. The additive model is a minimal model that gives this biologically reasonable behaviour.

#### Variable TCR sensitivity: The two-phase model

**Figure 4:**
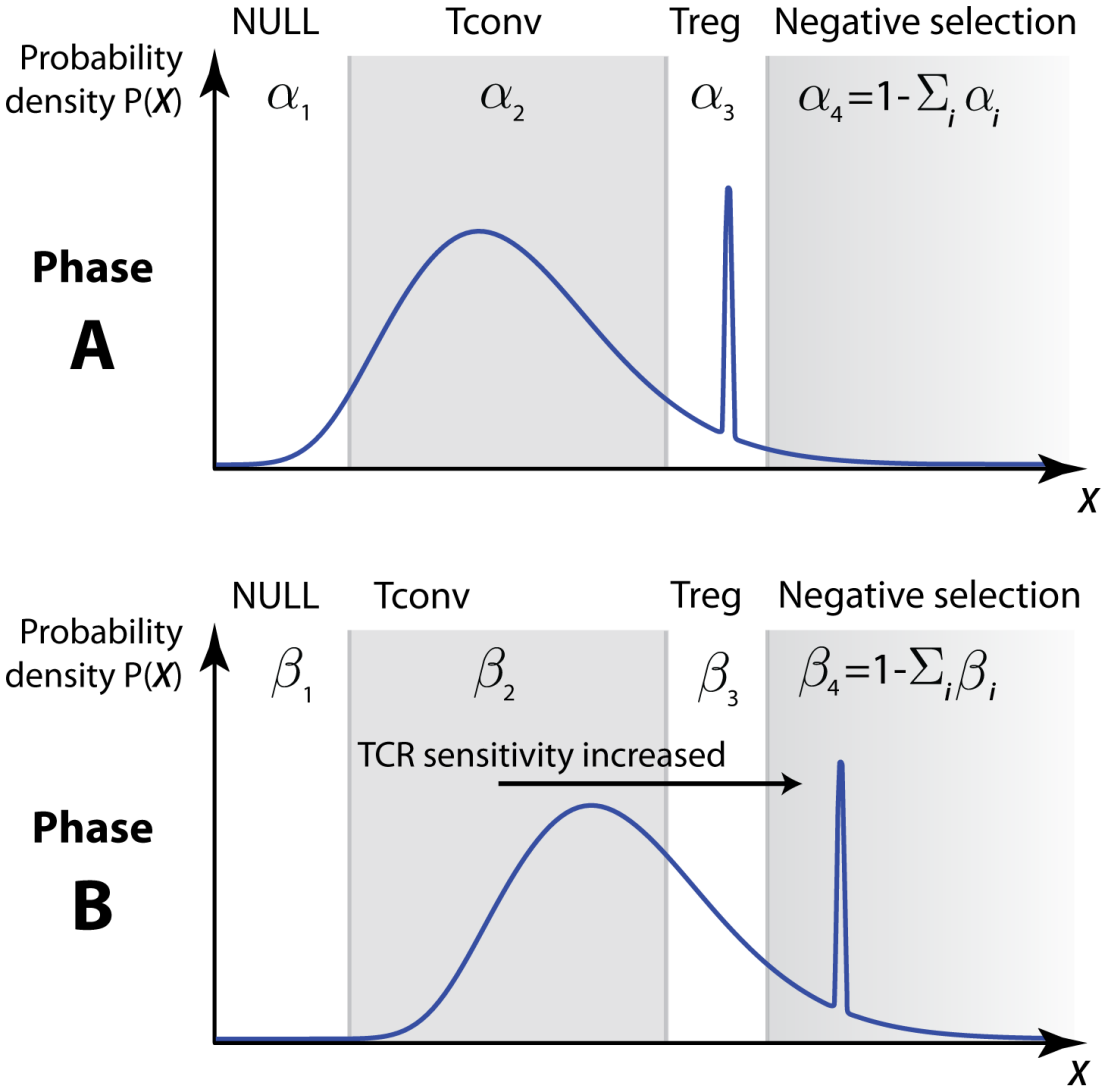
The two-phase model. In this instance, TCR sensitivity is assumed to increase during development, such that an encounter with the agonist ligand TIM delivers a signal that initiates T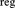 development early in selection (phase A) but causes deletion if encountered later (phase B).

This model is an extension of the one-hit (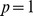) model that also allows the sensitivity of immature thymocytes to TCR stimulation to vary during maturation. For simplicity, the selection process is divided into two distinct phases, A and B, each with maximum number of interactions 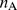 and 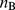. In each phase the TCR-pMHC encounters are divided into distinct selecting categories, as before, with probabilities 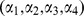 in the A-phase, where 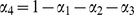, and probabilities 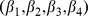 in the B-phase (Figure 4).

As above, we assume that a thymocyte participates in 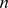 encounters at most. At any time during development a signal above the positive selection threshold 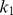 triggers T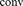 development; this decision can be superceded by a signal above the threshold 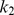 which triggers differentiation into the T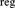 lineage; and a thymocyte can be negatively selected at any time by a signal greater than 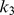.

The predictions of the model are independent of the order of the phases A and B. Here we discuss the situation in which TCR sensitivity increases during thymic development. In this scenario, 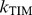 initially lies within the T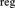-selecting region, meaning that an encounter with agonist in phase A initiates T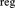 commitment and causes deletion in phase B (Figure 3). The changes in the quantities 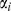 and 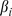 with TIM expression 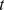 follow the form of equation 3, and we then express the probabilities of selection into each lineage as a function of 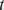 and the parameters 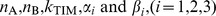. Details are in Text S2.

#### The choice of the distribution of signal strengths

In both models, a thymocyte's fate is determined by the maximum signal strength experienced over a large number of encounters. We assume the strength of a single TCR-pMHC binding is log-normally distributed. The strength of an encounter (the sum of 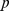 proximal TCR-pMHC interactions) is then also approximately log-normally distributed [55]. We choose the log-normal distribution because it is ubiquitous in cell biology, and arises naturally when a random variable is derived from multiplying random variables from arbitrary distributions – such as concentrations of different signalling molecules in signal transduction pathways. However the maximum value of a large sample drawn from any heavy-tailed distribution converges to the same (Fréchet) distribution [56], [57]. Each thymocyte is indeed expected to participate in a large number of encounters, and so our results hold for any heavy-tailed distribution of TCR-pMHC interaction strengths. Further, relative, not absolute, values of these signal strengths are key to the modelling of fate decisions and so we can set the mean of this distribution to be 1. The variance of the distribution is a free parameter which also does not influence our conclusions, but we discuss its influence on some parameter estimates in the Results.

#### Relating absolute peptide abundance to relative RNA expression

**Figure 5:**
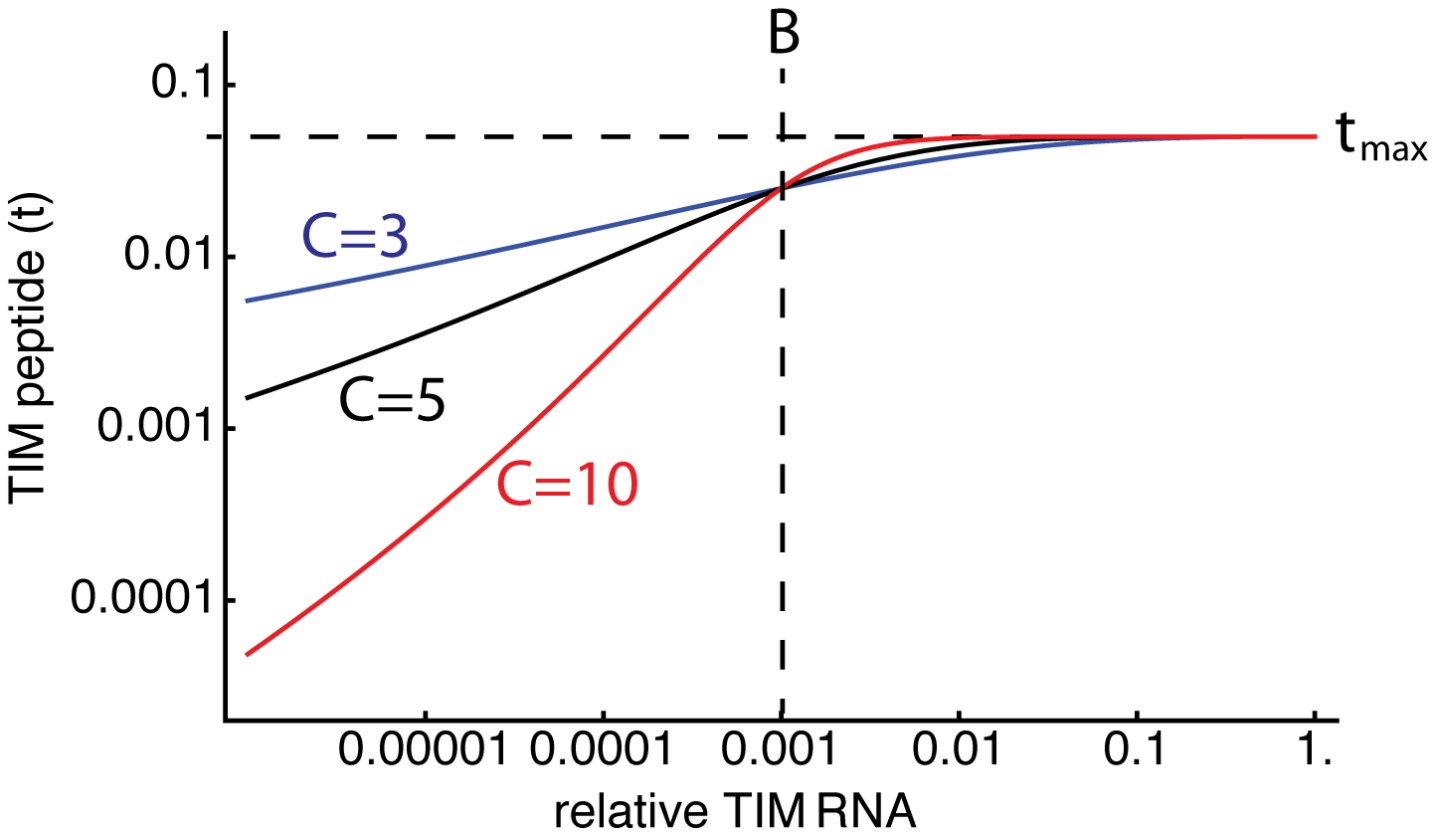
Relating TIM RNA to peptide abundance. We use equation 6 to connect the TIM peptide abundance 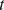 to the expression of TIM RNA relative to controls. Shown are three representative functions using different values of the breadth-parameter 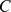, with location parameter 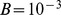 and saturating TIM abundance 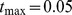.

We model agonist abundance 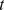 as the fraction of endogenous peptides replaced by the agonist peptide, while the measure of agonist abundance used in ref. [16] is the relative expression of TIM RNA compared to that in control thymi. The relationship between 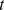 and TIM RNA is unknown, although we would expect it to increase monotonically. Further, a saturating level of TIM RNA is unlikely to achieve exclusive TIM expression (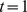), either due to competition for loading onto MHC from endogenous peptides and/or the presence of dendritic cells in the thymus that express endogenous but not TIM peptide MHC complexes. In the absence of more information we assume a sigmoid linear-log relation that is approximately linear at low TIM expression levels and saturates at 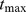,
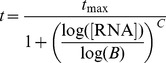
(6)where [RNA] is the TIM RNA expression level relative to controls and 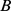 is the expression level at which 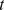 is half-maximum. 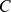 is a measure of the steepness of the function around 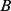 and is the slope of 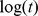 versus 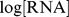 at low TIM expression levels (Figure 5). Despite our uncertainty in the relation between relative TIM RNA and 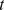, we will show that we can make robust statements regarding the ability of different models to describe the data.

### Parameter estimation

The key parameters of interest were the encounter size 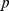 for the 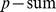 model, and the number of encounters in each phase in the two-phase model. Other parameters were estimated simultaneously, but several quantities were taken as inputs to the models because the data from [16] did not allow us to parameterise them directly. These were (i) the parameters specifying the relation between relative RNA expression and absolute peptide abundance; (ii) the distribution of signal strengths obtained by the AND TCR from randomly sampled self pMHC ligands; and (iii) the relation between selection probabilities and absolute cell numbers. First, we explored ranges of parameters defining the mapping function (equation 6); 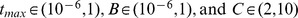. We chose to use this generic sigmoid dose-response curve given our ignorance of the mechanistic relation between RNA expression and peptide-MHC abundance on thymic epithelial cells. However, we were able to partially validate this choice of function, and the region of parameter space that we explored, using data from the study by Obst *et al.* [58]. They characterised the relation between the degree of activation of adoptively transferred AND 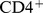 T cells and the relative TIM RNA expression on MHC class II-expressing cells, using a similar tetracycline-inducible expression system to that used in [16]. Their readout of immune activation was the fraction of AND cells that had divided 60 h following induction of TIM expression. Assuming this fraction is linearly related to peptide availability we used the data from Obst *et al.* to estimate the parameters of the mapping function (equation 6). We found that both the recruited fraction and an alternative measure of immune activation, the estimated *per capita* rate of recruitment into division, yielded mappings within the envelope of functions generated with our parameter ranges. These mappings also lay well within the 95% uncertainty envelope generated by the best-fitting parameters from our analysis of the data from [16]. For details, see Text S3, Figure S1 and Table S1. Second, we assumed the logarithm of the signal strength derived from a single AND-TCR endogenous-pMHC interaction is normally distributed with zero mean and unit variance. The scale of the distribution of signal strengths is arbitrary and its coefficient of variation does not influence our conclusions (see Results). Third, the models provide the probabilities of selection into the T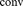 and T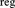 lineages and the data are absolute numbers of these populations in the thymus. We relate the numbers to probabilities through a scaling constant derived from the proportion of AND TCR cells that fail negative selection in control mice (Text S1).

#### Parameter estimation in the 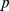-sum model

The 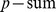 model is characterised by a further six parameters (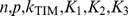) but three could be eliminated or constrained. First, because the AND TCR is strongly selecting we assumed that the probability of any one encounter falling below the positive selection threshold, 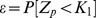, is small. Second, the parameter 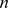 is the upper limit on the number of encounters made by a thymocyte during selection, and is expected to be large. Thymocytes move through the medulla and cortex at similar speeds (15 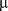
m/min and 10 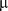
m/min, respectively) [59]. In the medulla, these speeds were shown to be associated with DC contacts at a rate of between 4 and 7 per hour, respectively. If we assume that additional contacts with TECs will contribute up to 50 contacts per hour, and that the time-spent in the thymus is between 5–10 days, then 10000 is a plausible upper bound on 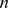. We used a conservative lower bound of 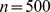. Thus the probability of all 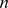 encounters falling below the positive selection threshold, 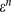, is vanishingly small and we set 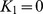. Further, for a given choice of 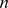 and 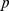, the thresholds for T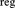 commitment (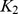) and negative selection (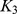) are determined by the observed probabilities of selection of conventional and regulatory T cells in control mice (Text S1). Selection in TIM transgenic mice using the 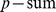 model is then described by three free parameters (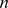, 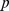, 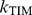). Only two of these can be identified uniquely, so we explored a discrete set of values of 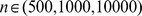 and for each used a maximum likelihood approach to identify values of 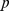 and 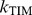, the signal strength derived from a single AND TCR contact with TIM agonist. The process was repeated across randomly sampled parameters characterising the mapping function. The residual sum of squares (RSS) and the Akaike information criterion (AIC), where with 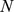 observations AIC = 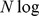
(RSS) up to an additive constant, were used to identify the best fitting parameter values. Approximate 95% confidence intervals were generated from the parameter sets that yielded AIC values within 2 units of the lowest value.

#### Parameter estimation in the two-phase model

The predictions of the two-phase model are determined by the TIM mapping function and the three parameters 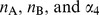 (Text S2). We varied 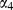, the probability that a randomly sampled pMHC in phase A will lead to negative selection, between 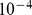 and 0.1, and used a maximum likelihood approach to identify 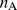 and 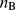. As above, the process was repeated for a wide range of mapping functions and AIC used to identify best-fitting parameter combinations and approximate 95% confidence intervals.

## Results

### Without TCR sensitivity varying during development, a model in which fate decisions derive from single TCR-pMHC contacts is unable to explain the data

The key features of the data are (i) T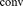 numbers decline monotonically with agonist expression and (ii) modest increases in agonist expression lead to an increase in the absolute number of AND T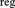, with numbers then decreasing at higher levels of TIM expression (Figure 1). Assuming there is a positive relationship between TIM peptide presentation (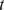) and relative TIM RNA expression, equation 4 shows that a model in which fate decisions are re-evaluated after single TCR-pMHC contacts (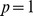) can describe the T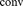 data, which falls progressively with 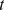.

**Figure 6:**
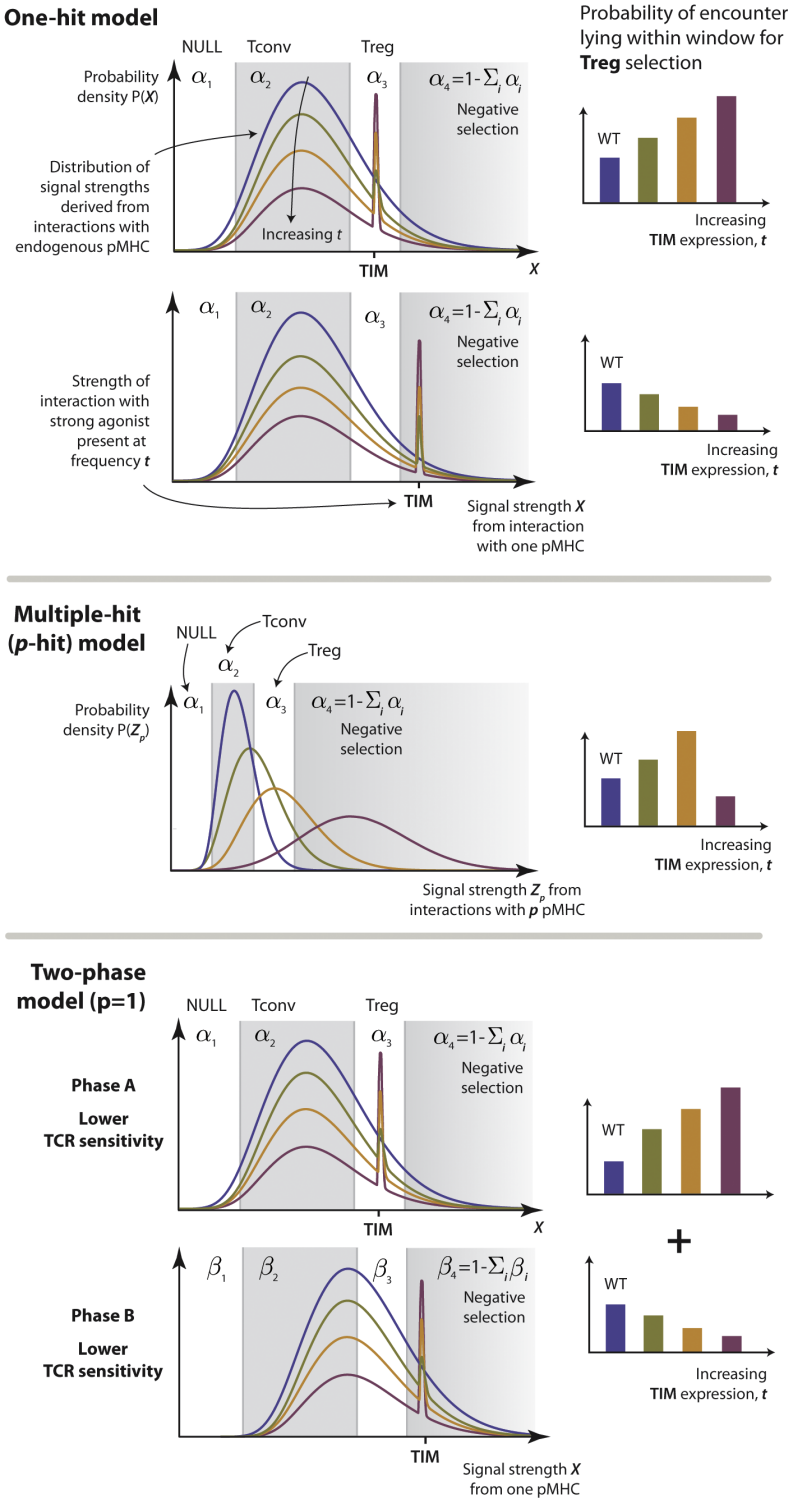
Modelling T 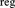 selection as a function of agonist abundance. **Upper panel** If TCR sensitivity remains static throughout thymic development, the simplest one-hit model fails to explain the rise and fall of T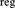 numbers with agonist expression, because the predicted probability of receiving a T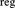 selecting signal either increases or decreases monotonically. **Middle panel**. Again with static thresholds, if encounters comprise 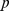 contacts with pMHC, and 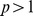, the distribution of signal strengths from each encounter 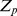 is smoother and shifts rightwards with TIM expression, first increasing then decreasing the probability 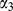 of triggering T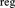 development, as required. **Lower panel**. The two-phase mode also explains the data and allows for encounters comprising single (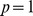, illustrated here) or multiple (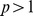) TCR-pMHC engagements to dictate fate. The trend in T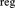 numbers arises from the balance between an increasing probability of receiving a T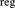 -selecting signal with agonist expression in the low-sensitivity phase, and a decreasing probability in the higher-sensitivity phase.

**Figure 7:**
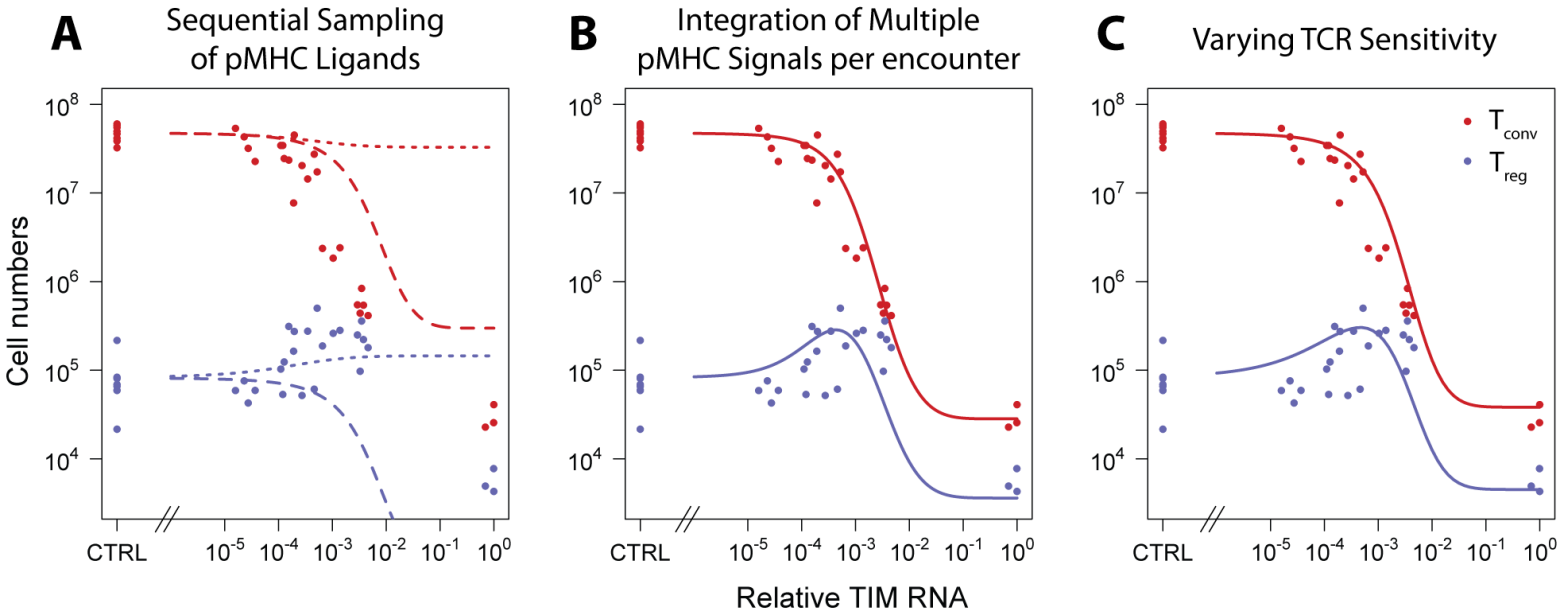
Model descriptions of the data. Representative descriptions of the data by the models. T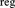 in blue, T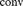 in red. **Panel A**; the one-hit model in which fate decisions are re-evaluated after single TCR-pMHC contacts. The dotted curves, 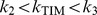; dashed curves, 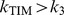. **Panel B**; the 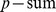 model; **Panel C**; the two-phase model with 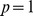.

However, we can see using a graphical argument (Figure 6, upper panel) that the 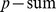 model with constant TCR sensitivity will only be able to capture the trend in T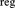 numbers if encounters comprise TCR signals integrated over multiple pMHC bindings (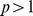). If the strength of an interaction between a single AND-TCR and agonist TIM (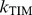) lies within the T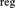-selecting range 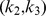, we would expect to see a monotonic increase in T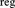 numbers with increasing agonist peptide expression; as agonist becomes more abundant, progressively more probability mass is contained within this area, boosting the probability of T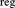 selection (Figure 5, upper panel; Figure 7A, dotted-blue curve). Here, the one-hit model predicts that the absolute increase in T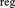 numbers is greater than or equal to the absolute decline in T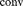 numbers. Conversely, if 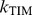 is above the threshold for negative selection, 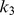, then we would predict a continuous decrease in T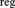 as agonist peptide becomes more abundant and increases the probability of deletion (Figure 5, upper panel; Figure 7A, dashed red curve). Neither of these trends are what is observed and so we rule out these scenarios. Finally, we can exclude the possibility that 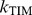 lies within the T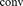 -selecting range; if 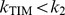, increasing TIM expression would then increase the probability of selection into T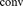, which we do not observe. Thus we can reject the simple one-hit model for selection of AND thymocytes.

### The data are consistent with a model in which fate decisions derive from integrating multiple TCR-pMHC encounters

Extending the argument above, to explain the rise and fall of T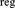 numbers with agonist peptide expression (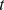) requires the probability mass within the T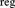-selecting region to increase then decrease with 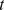. This becomes possible when thymocytes read multiple TCR-pMHC bindings simultaneously (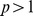). Qualitatively, this is because when 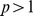, replacing an increasing fraction of endogenous peptides with TIM (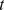) right-shifts the distribution of encounter strengths and, in contrast to the 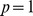 case, increases the probability of an encounter within both the T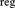 and negative-selection regions (Figure 3B). The probability contained below the T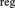 selection threshold falls with 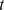, consistent with T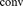 numbers falling; the probability 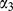 of an encounter occurring within the T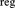 zone first increases then decreases with 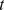, as required to explain the data; and the probability of negative selection continually increases (Figure 6, middle panel).

We explored this quantitatively and sought to identify the parameters of the 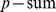 model from the data. They cannot all be identified uniquely. As described in Methods we took the approach of exploring a range of plausible parameters governing the function mapping RNA expression to endogenous peptide replacement by TIM, and a range of values of the maximum number of encounters, 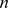.

Remarkably, all values of 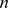 yielded equivalent descriptions of the data, and the encounter size 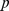 was highly insensitive to other parameters; it lay between 2 and 5 for all models, with best fitting value 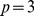, independent of 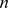. We also found that a range of mapping functions were able to describe the data equally well (Table S1). In particular, we predict that at maximum RNA expression, TIM replaces beween 0.1% and 12% of endogenous peptides. Representative fits to the data are shown in Figure 7. Panel A illustrates the failure of the one-hit model, with the best fit obtained by forcing 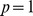. Panel B shows the fit achieved with the 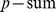 model with 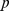 a free parameter.

The estimate of 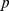 is also independent of the variance of the TCR-pMHC signal strength distribution 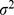. This also derives from the fact that the key quantities are just the probabilities 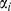 of interactions lying between the different thresholds 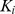. However these thresholds become increasingly spaced with 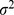 (that is, as the log-normal distribution becomes increasingly fat-tailed). The less heavy-tailed the distribution of signal strengths, the smaller is the window of affinity/avidity for triggering T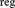 development with respect to the mean signal strength. Small increases in affinity can shift TCR signals from positively to negatively selecting [2], [3], and so if signal strength relates linearly to affinity or avidity [14], our model predicts that the distribution of encounter strengths with self may not be strongly heavy-tailed.

### The estimated encounter size 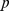 increases in the presence of null peptides

Anything between ten and a few hundred pMHC have been shown to be required for T cell activation (see for example, [60]) and as few as 3–5 for pMHC recognition by cytotoxic T cell effector function [61], although with the extent of TCR binding influencing the degree of activation [42]. However, data interpreted using the kinetic proofreading model suggest that multiple interactions with very weak ligands may not lead to activation at the whole cell level (see, for example, [40], [41], [62]). Therefore we wanted to test whether the low estimates of 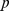 are an artefact of the assumption that every TCR-pMHC interaction generates a signal and so an encounter comprising 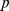 weak TCR-pMHC bindings might still lead to strong signalling.

To do this, we extended the 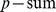 model such that only a fraction (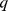) of self-peptides are capable of inducing a signal through the AND TCR, and the remaining fraction 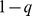 are classifed as null. This introduces a stochastic element to the number of TCR contributing to the signal from each encounter. We found that increasing the abundance of null ligands increases the estimated TCR engagements per encounter (Table S1). For example, we estimate the number of proximal TCR-pMHC engagements per encounter (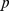) to be between 20–190 if 99% of peptides fail to trigger the TCR, and between 350–1000 when 99.9% of peptides are null. Intuitively, the increase in 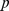 derives from the dilution of the information content of each encounter by the presence of null peptides. For each encounter to be a unit of sufficient information with which fate decisions can be triggered, the sample size 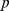 must increase in the presence of null interactions. As for the simpler model (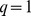) the estimate of 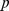 is also independent of the number of encounters, 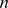.

Therefore, this extended model predicts that in the AND TCR system the expected number of productive TCR-peptide MHC interaction per encounter remains remarkably small (of the order 1). This is perhaps unsurprising, as low values of 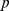 will allow thymocytes to discriminate between ligands with small differences in affinity.

### The two-phase model also explains the dependence of T_reg_ and T_conv_ on agonist expression

Next we explored the implications of a time-varying sensitivity of thymocytes to TCR stimulation during maturation. The two phase model, as described in Methods, extends the one-hit model to include time-varying TCR sensitivity. Its predictions are independent of the direction of variation, but to illustrate we assume an interaction with agonist leads to T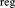 commitment during phase A early in development, but causes deletion in phase B when the same peptide is capable of inducing a stronger downstream TCR signal (Figure 4). Selection into the T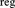 lineage is still possible in both phases; what changes between phase A and phase B is a right-shift in the distribution of signal strengths with respect to the selection thresholds. This shift in probabilities within the different fate-determining affinity ranges yields the required trends in T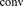 and T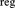 production with TIM expression (Figure 6, lower panel).

The details of parameter estimation for this model are in Methods and in Text S2. The unknowns are 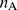, the number of encounters in phase A (Since 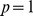, this is the maximum number of pMHC sampled in phase A), 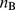, the number of encounters in phase B, and 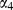, the probability that a randomly sampled pMHC in the low-sensitivity phase A will lead to negative selection.

**Figure 8:**
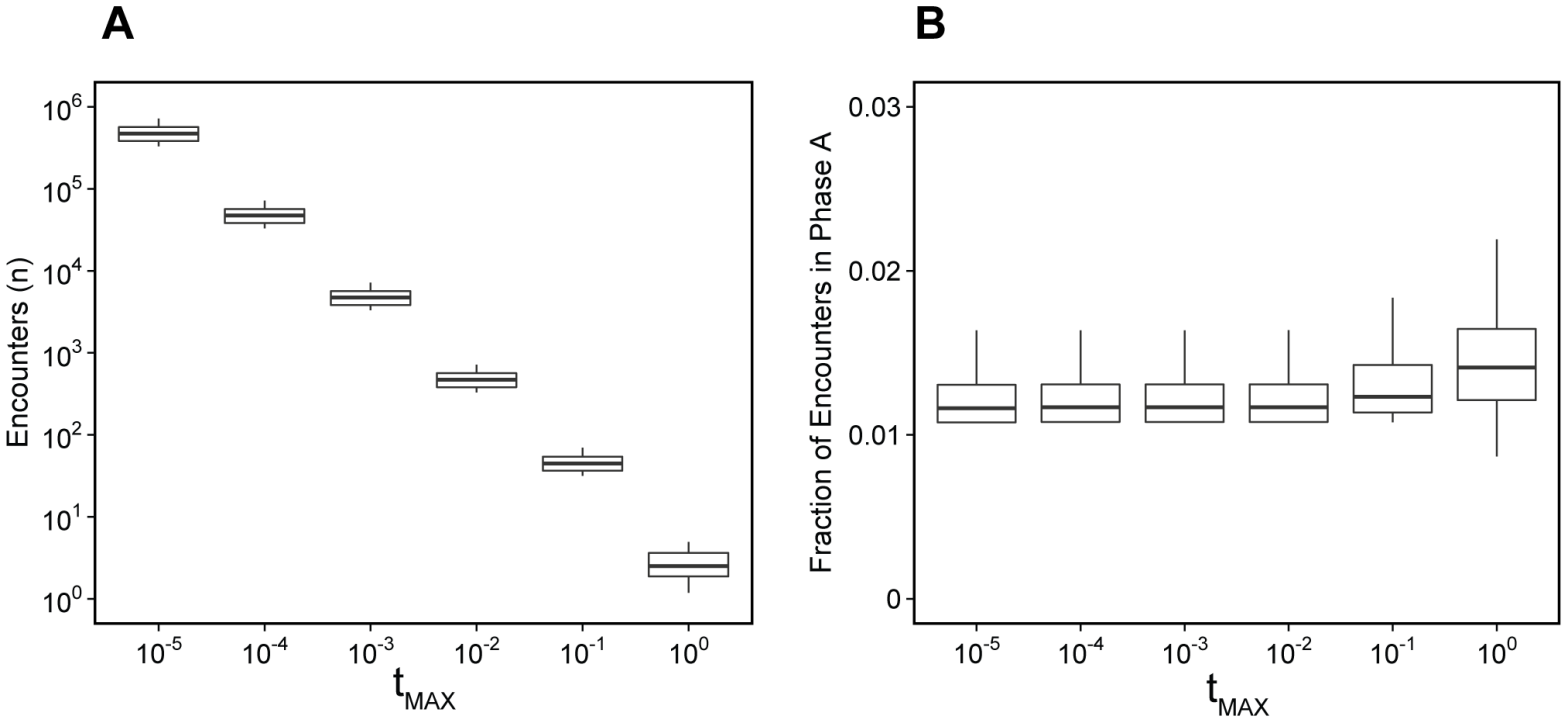
Two phase model. (A) Total number of encounters (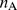
+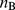) and (B) the proportion of all encounters that take place in phase A, for plausible ranges of 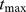, the proportion of endogenous peptides replaced by TIM at maximum RNA expression.

As for the 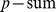 model, a range mapping functions described the data equally well. A representative fit using the two-phase model is shown in Figure 7C. We found a clear inverse relationship between the value of 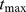 and the total number of encounters, 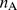
+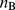 (Figure 8A). The model predicts that between 1–2% of encounters occur in the lower sensitivity phase (Figure 8B).

### Model discrimination: The two-phase model is required to explain the effect of partial and full agonists on T_reg_ selection

We have used data in which there is a profound loss of conventional T cells in the presence of relatively low frequency of agonist peptide, while T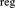 numbers are maintained and even initially increase with moderate increases in agonist frequency (Figure 1). These observations suggested the hypothesis that regulatory T cells are intrinsically more resilient to deletion by agonist peptides than conventional T cells [16]. Further, the study by Cozzo Picca *et al.* [15] showed that a partial agonist can induce deletion of conventional T cells but only an agonist could boost regulatory T cell generation. This led to a hypothesis that agonist peptide may deliver a qualitatively different signal that induces regulatory T cells.

We argue that neither of these hypotheses need be invoked. We have shown that both models can explain the first set of observations within a single affinity/avidity framework with different thresholds, without the need to assume differential susceptibilities of T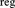 and T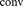 to deletion. Further, we can see immediately that the 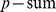 model will not explain the partial/full agonist observations in ref. [15]. Their observation that partial agonist increases the probability of deletion with no increase in T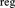 suggests that the presence of the partial agonist shifts the distribution of the sum of 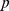 interactions far to the right of the wild-type distribution, such that the bulk of the distribution is contained above the negative selection threshold. It follows that strong agonist must push this distribution even further rightwards, and so the probability of signals lying within the T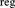-inducing zone must fall. This is inconsistent with the observed increase in T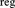 with agonist strength.

**Figure 9:**
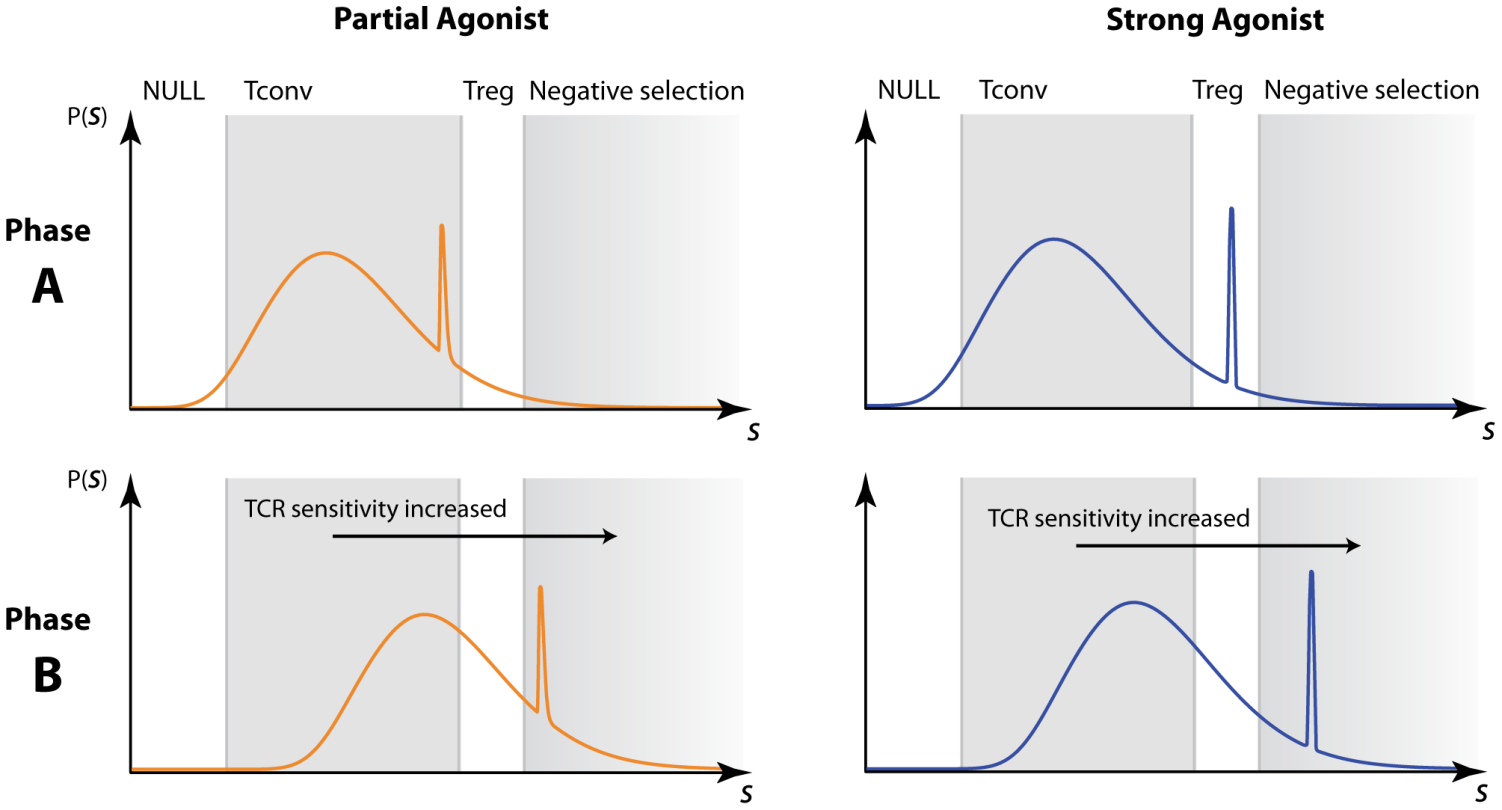
Using the two-phase model to explain the dependence of T 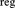 development on agonist strength. Partial agonist (left panels) may drive T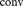 commitment in the early phase but induce deletion when TCR sensitivity is increased. In contrast, strong agonist (right panels) may drive T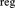 commitment early, and despite triggering deletion later, the net effect is still a net increase in T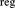 numbers. For clarity we illustrate here with the signal-strength distribution derived from 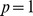, as in the basic two-phase model, but the argument holds also for a generalised two-phase model with encounters of size 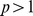 (Figure S2).

In contrast, the simple two-phase model can explain the effect (Figure 9). Assume that the partial agonist is not strong enough to induce T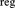 commitment in phase A when the TCR is relatively insensitive, but in the more sensitive phase B delivers a signal that lies above the negative selection threshold. Then the net effect of introducing a weak agonist is to increase deletion and have little effect on T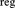 numbers, as is observed. In contrast, suppose the strong agonist triggers T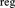 commitment when the TCR in phase A, but is negatively selecting in phase B (Figure 9, right hand columns). Then (i) expression of the strong agonist will always lead to a fall in conventional T cell numbers, and (ii) moderate levels of strong agonist, while increasing the overall probability of negative selection, can drive a net increase in T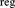 production by boosting the probability of receiving a T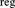-inducing signal in phase A.

Figure 9 illustrates this effect for the case 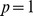, but the same argument holds for a generalisation of the two-phase model with 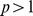 (Figure S2).

## Discussion

How does a thymocyte decide to become a conventional or regulatory T cell, or die, and when are these decisions most likely to take place? To address these questions, we have used experimental data to test models of how thymocytes interpret TCR interactions with self-peptide MHC to make fate decisions. We showed that the data cannot be explained with a model in which (from a given TCR's perspective) individual self-peptide MHC ligands are classed as positively selecting, negatively selecting or promoting T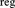 development, and in which a single engagement with a strong agonist pMHC is sufficient to influence cell fate. Instead we found stronger and roughly equivalent support for two alternative models, in which (i) a thymocyte bases decisions on TCR signals summed from multiple engagements with pMHC ligands, and/or (ii) a two-phase model in which TCR sensitivity alters during development, and so an engagement with the same pMHC ligand may lead to divergent outcomes at different stages of development. A robust prediction of the 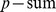 model is that the number of non-null TCR-self-peptide interactions per encounter that contribute to fate decisions is remarkably small. The two-phase model predicts that the initiation of differentiation into the T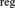 lineage is most efficient during a relatively short temporal window during which the TCR is less sensitive to stimulation. Importantly, the models express the probabilistic aspect of thymic selection that likely underlies the heterogeneity in lineage decisions within a clonotypic population.

### Explaining selection efficiency as a function of TCR affinity

We focused here on a system in which agonist availability could be manipulated. We can also use the models to make predictions regarding selection efficiency as a function of TCR affinity. Lee et al. [14] quantified the efficiency of T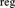 selection in the rat insulin promoter (RIP)-mOVA system for a range of 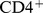 TCR clones with varying affinity for OVA. They found T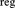 were generated across a broad range of reactivities and found an increase in T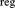 selection efficiency with increasing affinity for a self peptide. Both the 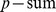 and variable-sensitivity models are able to explain these observations (Text S4 and Figure S3), but they make distinct predictions. The 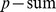 model predicts that the probability of the summed-proximal signals at each encounter exceeding the T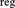 threshold is lower for weaker clones; but this probability remains constant throughout development. A variable-sensitivity model predicts that T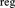 selection efficiency is determined by the duration of the window in which agonist contact triggers T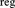 commitment. In an increasing-sensitivity scenario, clones that recognise OVA more weakly will take longer to reach a level of TCR sensitivity that can induce T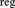, and so a prediction of the model is that lower-affinity TCR clones will commit to the T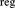 lineage later in development.

These models might be distinguished by manipulating thymocytes' TCR sensitivity, for example through altered expression of signalling proteins, at different stages of thymocyte development. The 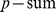 model predicts that the efficiency of T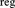 selection would be altered equally for all clones, whereas a model of increasing TCR sensitivity predicts that damping of TCR signalling later in development would most strongly reduce T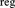 selection efficiency for clones with low affinity for self peptide.

### Robustness of results despite parameter uncertainties

We deliberately did not use model selection criteria to discriminate between models, in part because it is not possible to identify all parameters uniquely. Instead we identified regions of parameter space for each model that provided reasonable descriptions of the data. Importantly, the predicted values of the encounter size 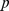 for different abundances of null peptides (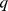) are insensitive to the remaining parameters (Table S1). We consider the 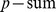 and two-phase models capable of describing the data equally well because they are able to capture the decline in T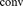 and increase then decrease in T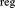 with TIM abundance. Both models capture this behaviour provided TIM abundance increases monotonically with RNA expression, which we expect to be the case. Further, model selection criteria are not required to reject the simplest one-hit model, nor to compare the abilities of the 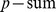 and two-phase models to describe the partial agonist observations.

### The effect of integrating many intermediate affinity binding events

One prediction of an avidity-based model is that thymocytes may be deleted if they interact simultaneously with several pMHC at moderate affinities. We believe this prediction is not necessarily unreasonable; such events may be an inevitable byproduct of a selection process that is inherently probabilistic and which can result in cells with identical TCR experiencing divergent fates.

### Generalisations of the two models

The two mechanisms we explore here are not mutually exclusive. We illustrated the two-phase model assuming that decisions are made based on single, rather than summed interactions with pMHC. However it can be generalised to an extended version of the 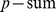 model in which encounters are interpreted differently as TCR sensitivity increases. This model will be able to explain the observations a least as well as the 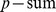 or two phase models, at the cost of extra parameters. Also, we illustrated the impact of increasing TCR sensitivity with a simple model that divided development into two discrete phases, while increases in TCR sensitivity are likely to be continuous. Modelling smooth changes in TCR sensitivity will introduce additional parameters but we expect such a model to yield qualitatively similar results. Importantly, our analysis does not exclude the possibility that T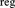 are more resistant to deletion and/or that qualitatively different signals are involved in T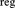 and T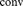 commitment; we simply show that these mechanisms need not be invoked to explain the observations.

### The nature of positively selecting signals

There is evidence that positive selection requires multiple low-affinity contacts with self-peptides in the thymus [63]–[65]. In contrast, in our model, a single encounter above the threshold 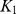 is sufficient for positive selection. We assumed this threshold is very low for the AND thymocytes, which are strongly selecting under normal conditions. These cells will presumably receive essentially continuous positively selecting signals. In the general case, and particularly for weakly signalling TCRs that are near the threshold for death by neglect, the threshold 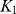 would need to be included in the parameter search and the model would be extended to include a memory of recent interactions; one possibility is a model in which levels of survival or fate-determining proteins are increased by TCR signalling but decay in its absence.

### The role of precursor frequency in limiting T_reg_ development

There is substantial evidence that increasing the frequency of a given clonal (single TCR specificity) population reduces its efficiency of selection and in particular the probability of being directed into the T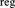 lineage [14], [22], [24]. Our models treat cells as independent entities and do not explicitly incorporate the possibility that competition between thymocytes of similar TCR specificities might influence the availability of selecting ligands. However one mechanism of competition can be represented quite straightforwardly in the models. If the strength of an encounter correlates with its duration, or perhaps increases the probability of internalisation of the pMHC ligand by the thymocyte, the TCR-specific cells will compete for and possibly sequester agonist and other high-avidity pMHC ligands. This will shift the apparent distribution of signal strengths leftwards towards lower avidity (and more available) interactions, reducing the probability of acquiring T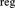-selecting signals. This model of competition for higher-avidity pMHC ligands may also explain the observation that the efficiency of T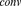 selection can increase with precursor frequency [22]. However it remains an open question whether competition for pMHC plays an important role in selection at physiological precursor frequencies.

### Cytokine requirements for T_reg_ development

Signalling through the IL-2 receptor is a requirement for T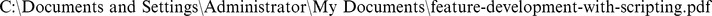 development [66]–[68]. It is thought that strong TCR signalling below the negative selection threshold may sensitise cells to IL-2, licensing progression towards the T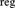 lineage. Whatever the precise role for IL-2, it must operate downstream of fate-determining signals if selection is governed by a hierarchy of TCR avidity thresholds. Nevertheless, if IL-2 is limiting it may provide an upper bound on the total rate of production of T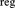, either by redirection of cells to the T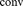 lineage or through loss. We argue however that competition for non-specific factors is unlikely to play a significant role in the system we are working with. First, the source of the IL-2 is unclear but we can reasonably suppose that IL-2 production in this system is independent of TIM expression. T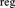 numbers increase with TIM at low expression levels, indicating that IL-2 cannot be limiting in this region (Figure 1). Similarly it cannot be limiting at higher TIM levels as T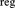 decrease. It remains possible that a capping of T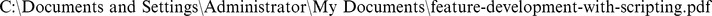 production through competition for IL-2 may be occuring in a small flat region near the peak in T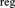 numbers, but competition for non-specific factors alone cannot explain the key aspects of T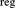 development we are attempting to describe.

### Predictions of the models regarding the timing of regulatory T cell commitment

Early neonatal thymectomy experiments suggested that the development of T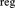 is delayed compared to conventional T cells [69]–[71]. A key T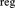 marker, the transcription factor Foxp3, is predominantly observed in the mature CD4 single positive stage of thymocyte development [72]. However, there may be a lag between initiation of T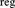 development and the stable expression of Foxp3; and it is possible that factors required for T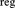 development such as IL-2 [66]–[68] or medullary thymic epithelial cells [73] may only be required later in the maturation process. Thus the timing of T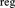 commitment remains unclear. The two models explored here make different predictions regarding this timing. The 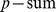 model suggests that diversion into the regulatory T cell lineage occurs with constant probability per encounter throughout selection. In contrast, the key prediction of the two-phase model is that the development of AND TCR T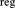 is triggered most efficiently within a relative short window during which thymocytes are relatively insensitive to TCR signalling. This window is estimated to span roughly 2% of the total pMHC ligands sampled, with the caveat that the two-phase model is an abstraction of what is more likely to be a temporally graded shift in sensitivity.

The two-phase model's predictions are identical whether the shorter, less sensitive phase occurs early or late in development. However, expression of the downstream TCR-signalling protein Zap70 increases progressively between the double positive (DP) and single positive stages of thymocyte development, and is associated with increasing sensitivity to TCR stimulation [28]; immature DP thymocytes display lower surface expression of TCRs, as compared to mature single positive (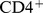 or 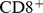) thymocytes, and TCR signalling may be actively inhibited in immature DPs [74]. Thymocytes' signalling environment may also change as they develop. Selection begins in the thymic cortex, where pMHC are encountered on cortical thymic epithelial cells, before cells migrate to the medulla where they encounter pMHC on medullary thymic epithelial cells and dendritic cells. It is thought that levels of co-stimulation and antigen presentation are generally lower in the cortex than in the medulla [75]–[77], suggesting that there may be an effective increase in TCR signalling during development. Using these observations, the two-phase model predicts that T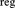 development begins predominantly, but not exclusively, during a short window at the earliest double positive stage of selection. Clearly, definitive experimental identification of when T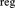 development is initiated will substantially increase our understanding of how thymocytes process information.

### Ligand discrimination — a role for time-varying TCR sensitivity in the thymus?

Reliable recognition and discrimination of self and nonself ligands requires both TCR sensitivity and specificity. Specificity will decrease as the number of pMHC integrated per encounter (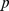) increases — when 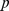 is large, many pMHC engagements are integrated at each encounter, and so the thymocyte is then just sampling the mean of the distribution of pMHC affinities, and information is lost. This may explain why the optimum values of 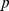 in the 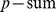 model are at the lower end of the reported numbers of pMHC engagements required for T cell activation; T cell activation may invole a relatively small number of informative TCR recognition events, together with many brief engagements with null or very low affinity peptide ligands. Our analysis of the 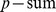 model places small lower bounds on the number of non-null TCR engagements per encounter; but the two-phase model explains the data by allowing even a single non-null pMHC recognition event to influence fate. We speculate that varying TCR sensitivity with time in the thymus may allow for increased specificity in self-nonself discrimination.

## Supporting Information

Figure S1
**Fitting the TIM mapping function to various readouts of immune activation derived from the data in Obst** ***et al.*** [**58**] **.** **Upper row**: Our proposed sigmoid mapping function yielded reasonable descriptions of three readouts of immune activation, all assumed to be proportional to peptide abundance on APC. **Lower panel**: we show the best fitting functions derived from the three measures superimposed on the 95% uncertainty envelope in mapping functions derived from the best-fitting parameters of the 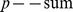 model. Assuming TIM abundance was proportional to the recruited fraction at 60 h (red curve) or to the per capita rate of recruitment (orange curve) yielded functions that lay well within this region.(EPS)

Figure S2
**Explaining the effect of partial and strong agonists on T** 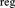 **selection efficiency.** Figure 9 in the main text illustrates how the observations of Cozzo Picca *et al.* [15] regarding the effect of partial and strong agonists on T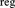 selection efficiency can be explained with the two phase model. This model can be extended to include multiple pMHC per encounter and with it a similar schematic can be used to explain the observations. As before, the temporal order of the low and high TCR sensitivity phases has no effect on our results; here we illustrate the argument for an increasing-sensitivity model. The presence of partial agonist may make T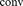 commitment far more likely than T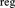 commitment in phase of lower TCR sensitivity (upper left panel). Increasing TCR sensitivity then may predominantly induce deletion (lower left panel). In contrast, strong agonist (right panels) may yield a higher probability of T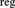 commitment when TCR sensitivity is lower but, as with a partial agonist, predominantly trigger deletion when TCR sensitivity increases. The net effect is an increase in T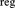 numbers as we shift from partial to strong agonist.(EPS)

Figure S3
**Alternative models of T** 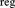 **selection for TCR clonotypes with varying sensitivity to endogenously expressed ovalbumin (OVA) peptide, as used in Lee** ***et al.*** **** [**14**] **.** In the 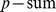 model (**upper panel**), each curve represents the distribution of summed signal strengths (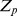) for TCRs with varying affinity for endogenously expressed OVA peptide. The probability that the summed signal from multiple pMHC contacts leads to T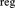 commitment is higher for TCR with higher affinity for OVA, and this probability remains constant for each encounter throughout development. In the varying-TCR sensitivity model (**lower panel**), each curve represents the strength of signal derived from a single contact with OVA for each TCR as a function of time. There is a window of susceptibility in which contact with an OVA pMHC will lead to T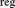 commitment; the probability of a contact with OVA is equal for all TCR, but the duration and timing of this window will vary for each TCR as a function of its initial ability to respond to OVA. (The blue line represents TCR with highest affinity for OVA; and orange represents TCR with the weakest affinity for OVA).(EPS)

Table S1
**Plausible combinations of parameters of the** 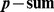 **model.** We used discrete combinations of 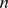, the maximum number of APC encounters made by a thymocyte, and 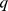, the fraction of endogenous peptides that are capable of inducing a TCR signal. Mean (minimum, maximum) values correspond to parameter combinations that described the data within 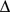
AIC
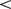
2 of the lowest AIC achieved for each (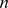,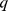) combination. 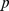 is the number of peptides contacted per APC encounter; 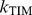 reflects the signal derived from a single TCR contact with agonist TIM (as a percentile of signal strengths derived from contacts with non-null endogenous peptides); 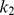 represents the minimal signal required for T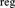 selection; 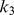 represents the minimal signal required for negative selection (percentile of signal strengths received per encounter (with functional and null endogenous peptides)); and 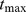, 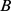 and 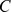 parameterise the mapping function from relative TIM RNA to peptide abundance.(PDF)

Text S1
**The calculation of the selection probabilities in the** 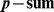 **model.**
(PDF)

Text S2 **The calculation of the selection probabilities in the two-phase model.**
(PDF)

Text S3 **Exploring the relation between relative TIM RNA expression and immune activation.**
(PDF)

Text S4
**Explaining variation in T** 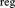 **selection efficiency with TCR affinity.**
(PDF)
