# Supplementary material for: Models of Self-Peptide Sampling by Developing T Cells Identify Candidate Mechanisms of Thymic Selection
Source: PLoS Comput Biol. 2013 Jul 25;9(7):e1003102. doi: 10.1371/journal.pcbi.1003102 (PMC3723501; doi:10.1371/journal.pcbi.1003102)
Supplement: Table S1 — Plausible combinations of parameters of the model. We used discrete combinations of , the maximum number of APC encounters made by a thymocyte, and , the fraction of endogenous peptides that are capable of inducing a TCR signal. Mean (minimum, maximum) values correspond to parameter combinations that described the data within AIC2 of the lowest AIC achieved for each (,) combination. is the number of peptides contacted per APC encounter; reflects the signal derived from a single TCR contact with agonist TIM (as a percentile of signal strengths derived from contacts with non-null endogenous peptides); represents the minimal signal required for T selection; represents the minimal signal required for negative selection (percentile of signal strengths received per encounter (with functional and null endogenous peptides)); and , and parameterise the mapping function from relative TIM RNA to peptide abundance. (PDF) [file pcbi.1003102.s004.pdf]

Table S1

n	q	p		k_{TIM}		k_2		k_3		$t_{\max}$		$\log_{10}(B)$		C	
500	0.001	465	(450, 540)	94.19	(80.24, 98.21)	99.76	(99.75, 99.77)	99.76	(99.75, 99.77)	0.0006	(0.0003, 0.001)	-3.1	(-3.5, -3)	5.0	(5, 5)
500	0.01	94	(35, 190)	93.59	(40.85, 98.91)	99.88	(99.67, 99.95)	99.88	(99.67, 99.95)	0.0986	(0.0032, 0.3162)	-3.4	(-3.5, -3)	5.4	(4, 7)
500	0.1	13	(9, 16)	94.69	(80.18, 98.96)	99.75	(99.61, 99.82)	99.75	(99.62, 99.82)	0.2214	(0.0316, 0.3162)	-3.3	(-3.5, -3)	5.7	(5, 7)
500	0.5	3	(2, 6)	99.63	(96.5, 99.92)	99.8	(99.6, 99.92)	99.8	(99.6, 99.92)	0.1116	(0.01, 0.3162)	-2.9	(-3.5, -2.5)	5.2	(5, 6)
500	0.9	3	(2, 3)	99.73	(99.57, 99.91)	99.66	(99.47, 99.71)	99.66	(99.47, 99.71)	0.0829	(0.0316, 0.1)	-2.9	(-3, -2.5)	5.0	(4, 6)
500	1	3	(3, 4)	99.6	(99.38, 99.81)	99.74	(99.74, 99.74)	99.74	(99.74, 99.74)	0.1000	(0.0562, 0.12)	-3.0	(-3, -3)	5.0	(4, 6)
1000	0.001	586	(350, 880)	75.47	(65.31, 86.28)	99.9	(99.83, 99.94)	99.9	(99.83, 99.94)	0.0080	(0.0032, 0.01)	-3.7	(-3.7, -3.7)	4.4	(4, 5)
1000	0.01	54	(20, 90)	99.12	(84.89, 99.61)	99.88	(99.68, 99.94)	99.88	(99.69, 99.94)	0.0072	(0.0032, 0.0316)	-3.0	(-3.7, -2.7)	5.1	(4, 6)
1000	0.1	9	(4, 21)	99.71	(82.42, 99.93)	99.93	(99.84, 99.98)	99.93	(99.84, 99.98)	0.0395	(0.0032, 0.3162)	-2.9	(-3.7, -2.7)	5.4	(4, 6)
1000	0.5	3	(2, 5)	99.85	(99.15, 99.97)	99.88	(99.8, 99.94)	99.88	(99.8, 99.94)	0.0527	(0.0032, 0.1)	-2.9	(-3.2, -2.7)	5.4	(4, 6)
1000	0.9	3	(2, 3)	99.92	(99.69, 99.97)	99.8	(99.73, 99.84)	99.8	(99.73, 99.84)	0.0440	(0.0032, 0.1)	-2.8	(-3.2, -2.7)	5.6	(5, 6)
1000	1	3	(3, 4)	99.87	(99.71, 99.97)	99.71	(99.7, 99.81)	99.71	(99.7, 99.81)	0.0505	(0.004, 0.1)	-2.9	(-3.2, -2.6)	5.2	(4, 6)
10000	0.001	450	(350, 650)	87.7	(84.28, 93.13)	99.98	(99.98, 99.99)	99.98	(99.98, 99.99)	0.0083	(0.0032, 0.01)	-3.7	(-3.7, -3.7)	5.8	(5, 6)
10000	0.01	51	(20, 130)	98.99	(77.48, 99.73)	99.98	(99.97, 99.99)	99.98	(99.97, 99.99)	0.0188	(0.0032, 0.1)	-3.5	(-3.7, -3)	4.9	(4, 6)
10000	0.1	5	(3, 15)	98.06	(75.9, 98.65)	99.99	(99.97, 100)	99.99	(99.97, 100)	0.0251	(0.01, 0.1778)	-3.1	(-3.7, 2.5)	5.2	(4, 6)
10000	0.5	4	(2, 10)	99.94	(97.31, 99.99)	99.98	(99.96, 99.99)	99.98	(99.96, 99.99)	0.0460	(0.0032, 0.3162)	-3.1	(-3.5, -2.7)	5.0	(4, 6)
10000	0.9	3	(2, 5)	99.97	(99.57, 99.99)	99.98	(99.97, 99.99)	99.98	(99.97, 99.99)	0.0322	(0.0032, 0.1)	-3.0	(-3.5, -2.7)	5.2	(4, 6)
10000	1	3	(2, 5)	99.98	(99.59, 99.99)	99.98	(99.98, 99.99)	99.98	(99.98, 99.99)	0.0251	(0.001, 0.1)	-2.8	(-3.5, -2.5)	5.1	(4, 6)
