# Supplementary material for: Models of Self-Peptide Sampling by Developing T Cells Identify Candidate Mechanisms of Thymic Selection
Source: PLoS Comput Biol. 2013 Jul 25;9(7):e1003102. doi: 10.1371/journal.pcbi.1003102 (PMC3723501; doi:10.1371/journal.pcbi.1003102)
Supplement: Text S1 — The calculation of the selection probabilities in the model. (PDF) [file pcbi.1003102.s005.pdf]

Text S1: The p-sum model

Let Z_p be the signal strength obtained from one T cell encounter, comprising p self-peptide MHC ligands. Let the thresholds K_1, K_2, and K_3 be the minimum signal strengths required for T_{conv} commitment, T_{reg} commitment and negative selection, respectively; and let n be the maximum number of encounters during development (a cell may die through negative selection before these are completed). Then

$$P[T_{conv}] \simeq P[Z_p \leq K_2]^n - P[Z_p \leq K_1]^n \quad \text{(S1-1)}$$

$$P[T_{reg}] \simeq P[Z_p \leq K_3]^n - P[Z_p \leq K_2]^n. \quad \text{(S1-2)}$$

In the absence of TIM expression, $t = 0$, Z_p is the sum of p independent lognormally distributed random variables $X_i, i = 1 \ldots p$, with unknown variance but mean set to unity without loss of generality. In the presence of TIM expressed at frequency t, the X_i are drawn from the mixed distribution $t\,\delta(k_{\mathrm{TIM}}) + (1 - t)\,\mathrm{LogNorm}(0, \sigma^2)$, where $\delta(.)$ is the Dirac delta function.

We assume that the probability of failing positive selection ($P[Z_p \leq K_1]^n$) is negligible for AND specific thymocytes (see Methods) and let $X_i \sim \mathrm{LogNorm}(0, \sigma^2 = 1)$. The model provides probabilities of selection into each lineage as a function of the selection thresholds and other parameters, while the data provide the numbers of cells in the two lineages. The model and data are connected here by a scaling constant determined from the unknown proportion f of AND cells failing negative selection in the controls (that is, in the absence of cognate pMHC). Given n, p, f, the parameters characterising the lognormal distribution of signal strengths, and the mean observed T_{conv} and T_{reg} numbers in control animals, equations S1-1 and S1-2 yield K_2 and K_3.

The negative selection probability in controls, f, is unknown but approximately 50×10^6 cells in the thymi of control animals possessed the AND TCR (Figure 4B in ref. [1]) which is roughly half the number of T cells found in a typical B6 mouse thymus. This strongly suggests that negative selection of AND TCR occurs at a low rate in the absence of TIM agonist. (As an aside, because the number of encounters n is large, and the probability of negative selection is $f = 1-(\mathrm{Prob}(Z_p < K_3))^n$, then to avoid near-complete deletion of AND TCRs through negative selection in the absence of agonist requires the negative selection threshold K_3 to be far into the tail of the distribution of Z_p derived from endogenous peptides, as seen in Table S1.) Intuitively, the information constraining the encounter size p derives from the fold changes in selection probabilities as TIM agonist abundance increases, and should not depend strongly on the baseline rate of negative selection in the absence of agonist. To validate this, we performed analyses for f = 0.1, 0.5 and 0.9; p and its associated confidence interval were independent of f.

References

[1] van Santen HM, Benoist C, Mathis D (2004) Number of T reg cells that differentiate does not increase upon encounter of agonist ligand on thymic epithelial cells. J Exp Med 200: 1221-30.
