# Supplementary material for: Models of Self-Peptide Sampling by Developing T Cells Identify Candidate Mechanisms of Thymic Selection
Source: PLoS Comput Biol. 2013 Jul 25;9(7):e1003102. doi: 10.1371/journal.pcbi.1003102 (PMC3723501; doi:10.1371/journal.pcbi.1003102)
Supplement: Text S2 — The calculation of the selection probabilities in the two-phase model. (PDF) [file pcbi.1003102.s006.pdf]

Text S2: The Two Phase Model

We propose that an interaction with agonist peptide present at frequency t triggers T_{reg} development during Phase A but deletion in Phase B, when the same peptide is capable of inducing a stronger downstream TCR signal. The adjusted probabilities of a TCR-pMHC encounter within the four selecting regions are $((1-t)\alpha_1, (1-t)\alpha_2, (1-t)\alpha_3 + t, (1-t)\alpha_4)$ in phase A, and $((1-t)\beta_1, (1-t)\beta_2, (1-t)\beta_3, (1-t)\beta_4 + t)$ in phase B. Each cell has up to $n_{\rm A}$ and $n_{\rm B}$ encounters in the respective phases.

The probabilities of selection into each lineage as a function of agonist frequency t are then

$$\begin{aligned}
P[\mathrm{T_{conv}}(t)] &= (1-t)^{n_{\rm A}+n_{\rm B}}(\alpha_1+\alpha_2)^{n_{\rm A}}(\beta_1+\beta_2)^{n_{\rm B}} - (1-t)^{n_{\rm A}+n_{\rm B}}\alpha_1^{n_{\rm A}}\beta_1^{n_{\rm B}} \\
P[\mathrm{T_{reg}}(t)] &= [(1-t)(1-\alpha_4)+t]^{n_{\rm A}}(1-t)^{n_{\rm B}}(1-\beta_4)^{n_{\rm B}} \\
&\quad -(1-t)^{n_{\rm A}+n_{\rm B}}(\alpha_1+\alpha_2)^{n_{\rm A}}(\beta_1+\beta_2)^{n_{\rm B}} \\
&= ((1-t)^{n_{\rm A}}(1-\alpha_4)^{n_{\rm A}} + [(1-t)(1-\alpha_4)+t]^{n_{\rm A}} - (1-t)^{n_{\rm A}}(1-\alpha_4)^{n_{\rm A}}) \\
&\quad \times(1-t)^{n_{\rm B}}(1-\beta_4)^{n_{\rm B}} - (1-t)^{n_{\rm A}+n_{\rm B}}(\alpha_1+\alpha_2)^{n_{\rm A}}(\beta_1+\beta_2)^{n_{\rm B}}, \\
&= (1-t)^{n_{\rm A}+n_{\rm B}}(1-\alpha_4)^{n_{\rm A}}(1-\beta_4)^{n_{\rm B}} - (1-t)^{n_{\rm A}+n_{\rm B}}(\alpha_1+\alpha_2)^{n_{\rm A}}(\beta_1+\beta_2)^{n_{\rm B}} \\
&\quad +(1-t)^{n_{\rm B}}(1-\alpha_4)^{n_{\rm A}}(1-\beta_4)^{n_{\rm B}}\left[\left(1-t+\frac{t}{1-\alpha_4}\right)^{n_{\rm A}} - (1-t)^{n_{\rm A}}\right].
\end{aligned}$$

By definition,

$$\begin{aligned}
P[\mathrm{T_{conv}}(\mathrm{WT})] &= (\alpha_1+\alpha_2)^{n_{\rm A}}(\beta_1+\beta_2)^{n_{\rm B}} - \alpha_1^{n_{\rm A}}\beta_1^{n_{\rm B}}, && \text{(S2-1)} \\
P[\mathrm{T_{reg}}(\mathrm{WT})] &= (1-\alpha_4)^{n_{\rm A}}(1-\beta_4)^{n_{\rm B}} - (\alpha_1+\alpha_2)^{n_{\rm A}}(\beta_1+\beta_2)^{n_{\rm B}} && \text{(S2-2)}
\end{aligned}$$

and if we assume that failure to positively select is negligible for AND^+ T cells (*i.e.* $\alpha_1^{n_{\rm A}}\beta_1^{n_{\rm B}} \ll 1$), then $(1-\alpha_4)^{n_{\rm A}}(1-\beta_4)^{n_{\rm B}} \approx P[\mathrm{T_{conv}}(\mathrm{WT})] + P[\mathrm{T_{reg}}(\mathrm{WT})]$. Thus six unknown parameters $\beta_{1,2,3}$ and $\alpha_{1,2,3}$ are removed from the calculation, and the model becomes:

$$\begin{aligned}
P[\mathrm{T_{conv}}(t)] &= (1-t)^{n_{\rm A}+n_{\rm B}}P[\mathrm{T_{conv}}(\mathrm{WT})] \\
P[\mathrm{T_{reg}}(t)] &= (1-t)^{n_{\rm A}+n_{\rm B}}P[\mathrm{T_{reg}}(\mathrm{WT})] + \\
&\quad (1-t)^{n_{\rm B}}(P[\mathrm{T_{conv}}(\mathrm{WT})] + P[\mathrm{T_{reg}}(\mathrm{WT})])\left[\left(1-t+\frac{t}{1-\alpha_4}\right)^{n_{\rm A}} - (1-t)^{n_{\rm A}}\right].
\end{aligned}$$

So we obtain a prediction for conventional and regulatory T cell numbers as a function of agonist frequency, number of interactions in each phase, the probability of encountering a negatively selecting ligand in Phase A, and wild-type conventional and regulatory T cell numbers. These parameters are insensitive to the scaling constant derived from the proportion of AND TCR that are negatively selected in controls (see Text S1). The model's predictions do not depend on the temporal order of phase A and phase B, and so allow for TCR sensitivity to either increase or decrease with time during development.
