# Supplementary material for: Models of Self-Peptide Sampling by Developing T Cells Identify Candidate Mechanisms of Thymic Selection
Source: PLoS Comput Biol. 2013 Jul 25;9(7):e1003102. doi: 10.1371/journal.pcbi.1003102 (PMC3723501; doi:10.1371/journal.pcbi.1003102)
Supplement: Text S3 — Exploring the relation between relative TIM RNA expression and immune activation. (PDF) [file pcbi.1003102.s007.pdf]

Text S3: Exploring the relation between relative TIM RNA expression and immune activation

As described in Methods, we wanted to validate our choice of the function $t(R)$ mapping TIM RNA expression (R, relative to the housekeeping gene hypoxanthine phosphoribosyltransferase) to TIM peptide abundance t. To approach this we used data from Obst *et al.* [1], who measured the fraction of specific cells recruited into an immune response by 60h, which we denote f, for varying levels of TIM RNA expression R.

Their data provides $f(R) = \tilde{f}(t(R))$, and we require $t(R)$. However, we lack a mechanistic model of any readout of immune activation and peptide availability on APCs; thus the form of $\tilde{f}(t)$ is unknown. We wished to validate our sigmoid form for $t(R)$, and the estimated parameters:

$$t(R) = \frac{t_{\max}}{1 + (\log_{10}(R - B))^C}. \tag{S3-1}$$

To do this we first assumed that the recruited fraction f was simply directly proportional to TIM abundance, $f = at$. Thus

$$f = \frac{at_{\max}}{1 + (\log_{10}(R - B))^C}. \tag{S3-2}$$

We then estimated the parameters B and C and the compound parameter $at_{\max}$ from the data presented in Figure 2 in ref. [1]. The best fitting model appeared to describe the data well (Figure 1, upper left panel). We then explored two alternative measures of immune activation, both assumed proportional to TIM abundance, and obtained using the original CFSE-staining data. One measure was the *per capita* rate of recruitment into the first division, r. Here r (in units of hours^{-1}) can be estimated simply from the recruited fraction at 60h, which is $q = 1 - \exp(-r \times 60)$. The other was the mean division number at 60h, d, defined as

$$d = \frac{\sum_i i x_i 2^{-i}}{\sum_i x_i 2^{-i}} \tag{S3-3}$$

where the x_i are the proportions of cells in each CFSE peak at 60h. Again, the parameters B, C and $at_{\max}$ were estimated for each of these measures. Eqn. S3-2 yielded reasonable descriptions of all three datasets (Figure S1, upper three panels).

We were then able to compare the mapping function estimated from the data in Obst *et al.* with the range of best-fitting mapping functions derived in the main text using the data from van Santen *et al.*. Note that $t_{\max}$ itself cannot be estimated from these new data because it is confounded by the proportionality constant a. However we could compare the functions when expressed as proportions of the maximum TIM expression, $1/(1 + (\log_{10}(R - B))^C)$ (Figure S1, lower panel).

We see that if one assumes peptide availability t is proportional to the fraction of specific cells recruited within a given time interval, or with the probability of recruitment per cell per unit time, the dose-response function mapping RNA to t agrees well with that derived from manipulations of peptide abundance in the thymus.

Mean division number yielded poor agreement. This is perhaps unsurprising given that recruitment is likely very strongly influenced by antigen availability, but that average rates of proliferation are likely influenced by multiple other factors. Under reasonable assumptions, then, the data of Obst *et al.* provide a partial validation of the TIM mapping function we identify in the main text.

References

[1] Obst R, van Santen HM, Mathis D, Benoist C (2005) Antigen persistence is required throughout the expansion phase of a CD4(+) T cell response. J Exp Med 201: 1555-65.
