# Supplementary material for: Models of Self-Peptide Sampling by Developing T Cells Identify Candidate Mechanisms of Thymic Selection
Source: PLoS Comput Biol. 2013 Jul 25;9(7):e1003102. doi: 10.1371/journal.pcbi.1003102 (PMC3723501; doi:10.1371/journal.pcbi.1003102)
Supplement: Text S4 — Explaining variation in T selection efficiency with TCR affinity. (PDF) [file pcbi.1003102.s008.pdf]

Text S4: Explaining variation in T_{reg} selection efficiency with TCR affinity

Here we describe how the p-sum and increasing-sensitivity models are able to explain the observations of Lee *et al.* [1], who found T_{reg} were generated across a broad range of reactivities to an OVA peptide and found an increase in T_{reg} selection efficiency with increasing affinity for OVA.

The p-sum model

According to the p-sum model, the signal strength obtained from each thymocyte encounter, comprising p self-peptide MHC ligands, Z_p, is defined as: $\sum_{i=1}^{p} t\,\delta(k_{\mathrm{OVA}}) + (1-t)\,\mathrm{LogNorm}(0,\sigma^2)$; where $\delta(.)$ is the Dirac delta function; k_{OVA} is the strength of signal derived from a single TCR:OVA peptide interaction; and t is the fraction of thymic MHC ligands that are presenting the OVA peptide. If we assume, parsimoniously, that (i) k_{OVA} increases for TCRs with increasing affinity for OVA; and (ii) the level of stimulation received from endogenous peptides is unchanged between TCRs with different affinity for OVA, then, in a system in which OVA is presented intra-thymically, the distribution of summed-signal strengths (Z_p) will shift furthest rightwards for TCR transgenics that recognise OVA with the highest affinity (Figure S3).

As a result, increased affinity for OVA will invariably lead to an increase in the probability of a negatively selecting encounter, and a decrease in the probability of null or T_{conv}-selecting encounters. Furthermore, it can be shown that there exists a window of affinities for OVA in which the probability of a T_{reg} selecting encounter will increase sufficiently to give a net increase probability of T_{reg} commitment with increasing affinity, despite the concomitant increase in the probability of a negatively selecting event. For TCR with affinity for OVA that exceeds the upper bound on this region, the probability distribution will shift sufficiently far towards negatively selecting events that the net probability of T_{reg}selection (and the T_{reg}:T_{conv} ratio) will decrease.

The variable-TCR-sensitivity model

According to the two-phase model, contact between a TCR and its cognate antigen will either lead to T_{reg} or negative selection. In order to consider the selection of TCRs with weaker affinity for OVA, we extend the two-phase model to include three phases such that an encounter with OVA might lead to (i) T_{conv} selection (during phase 1 of development); (ii) T_{reg} selection (phase 2); or (iii) negative selection (phase 3). The predictions of these three phase model are independent of the direction of change in sensitivity, but in Figure S3 we illustrate a continuous increase in TCR sensitivity as reflected by the avidity for OVA peptide over time during development, relative to selection thresholds. It is assumed that OVA-specific DO11 TCR will behave in a similar manner to the MCC-specific AND TCR (that is, encounters with OVA lead to T_{reg} or negative selection only); while (i) an encounter with OVA will leader to weaker signalling in TCR with lower affinity for the peptide, introducing the possibility of receiving T_{conv} selection signals (phase 1 events) for sufficiently weak TCRs; (ii) all clonotypes increase TCR sensitivity during thymic development; and (iii) the hierarchy of TCR affinities for a OVA is maintained during development, despite this tuning of TCR sensitivity.

The selection of TCRs with a broad range of affinities for OVA is modelled using parallel signal strength functions (Figure S3). For any function in which the initial increase in TCR sensitivity is convex (increasing

at an increasing rate), the number of encounters involving a contact with OVA peptide that lead to T_{reg} selection will positively correlate with affinity (Figure S3). Interestingly, the three-phase model predicts that T_{reg} selection is possible across a broad range of TCR affinities for a self-antigen despite selection thresholds that only permit T_{reg}selection within a narrow range of avidities.

However, lower-affinity TCR will (i) take longer to drive their TCR sensitivity to a level at which an encounter with OVA peptide will lead to a T_{reg}-selecting event; and (ii) the duration of this window of susceptibility to T_{reg} selection will decrease, resulting in decreased net T_{reg} selection efficiency. In contrast, in the p-sum model the probability that an encounter triggers T_{reg} commitment is higher for TCR with higher affinity for OVA, but this probability remains constant throughout development.

References

[1] Lee HM, Bautista JL, Scott-Browne J, Mohan JF, Hsieh CS (2012) A broad range of self-reactivity drives thymic regulatory T cell selection to limit responses to self. Immunity 37: 475-86.
